# Proteolytic Characteristics of Cathepsin D Related to the Recognition and Cleavage of Its Target Proteins

**DOI:** 10.1371/journal.pone.0065733

**Published:** 2013-06-20

**Authors:** Huiying Sun, Xiaomin Lou, Qiang Shan, Ju Zhang, Xu Zhu, Jia Zhang, Yang Wang, Yingying Xie, Ningzhi Xu, Siqi Liu

**Affiliations:** 1 Beijing Institute of Genomics, Chinese Academy of Sciences, Beijing, China; 2 University of Chinese Academy of Sciences, Beijing, China; 3 Beijing Protein Innovation, Beijing, China; 4 Laboratory of Cell and Molecular Biology, Cancer Institute and Cancer Hospital, Chinese Academy of Medical Sciences and Peking Union Medical College, Beijing, China; California Institute of Technology, United States of America

## Abstract

Cathepsin D (CD) plays an important role in both biological and pathological processes, although the cleavage characteristics and substrate selection of CD have yet to be fully explored. We employed liquid chromatography-tandem mass spectrometry (LC-MS/MS) to identify the CD cleavage sites in bovine serum albumin (BSA). We found that the hydrophobic residues at P1 were not only a preferential factor for CD cleavage but that the hydrophobicity at P1’ also contributed to CD recognition. The concept of hydrophobic scores of neighbors (HSN) was proposed to describe the hydrophobic microenvironment of CD recognition sites. The survey of CD cleavage characteristics in several proteins suggested that the HSN was a sensitive indicator for judging the favorable sites in peptides for CD cleavage, with HSN values of 0.5–1.0 representing a likely threshold. Ovalbumin (OVA), a protein resistant to CD cleavage in its native state, was easily cleaved by CD after denaturation, and the features of the cleaved peptides were quite similar to those found in BSA, where a higher HSN value indicated greater cleavability. We further conducted two-dimensional gel electrophoresis (2DE) to find more proteins that were insensitive to CD cleavage in CD-knockdown cells. Based on an analysis of secondary and three-dimensional structures, we postulated that intact proteins with a structure consisting of all α-helices would be relatively accessible to CD cleavage.

## Introduction

Cathepsins are a class of lysosomal proteases that play important roles in proteolysis during physiological processes. They are reportedly involved in a number of diseases, such as cancer [1], [2], [3], atherosclerosis [4], arthritis [5] and neurodegenerative diseases [6], [7]. Several cathepsins can function outside of cells. For example, cathepsins B, D and L are able to cleave proteins in the extracellular matrix (ECM), including collagen [8], fibronectin [9], proteoglycans [10] and laminin [9], [11], and are considered to represent causal factors in tumor invasion and metastasis. Therefore, identification of the native substrates and cleavage sites of cathepsins is necessary to understand their physiological and pathological roles.

Cathepsin D (CD) is an aspartic endoprotease that is widely distributed in mammalian cells. Because of dual locations of CD, being either located in organelles such as in the cytoplasm, lysosomes and phagosomes, or secreted into the ECM, it participates in a number of physiological processes, including cell proliferation [12], apoptosis [13], [14], senescence [15] and tissue homeostasis [16]. CD is also known to take part in various pathological processes; it is likely involved in cancer development as well as metastasis [12], [17], atherosclerosis [18], [19] and Alzheimer’s disease [20], [21]. Like other cathepsins, CD recognizes its substrate with a relatively low selectivity. Nevertheless, it does not function on some proteins under certain circumstances, such as hen egg white lysozyme and ovalbumin (OVA) [22], [23]. In the field of CD research, it remains to be systematically evaluated which cleavage sites of target proteins, particularly for native forms, are favored for CD action.

Previous investigations of CD cleavage sites were initiated using synthetic peptides and medium-sized natural peptides, which revealed that the amino acid residues at P2, P2’, P3 and P3’ exerted some influence on the susceptibility to CD cleavage [24] and the hydrophobic residues co-occupying P1 and P1’ favored CD attack [25]. As the spatial structures of proteins are different from peptides, the information on cleavage sites favored by CD derived only from peptides is not sufficiently convincing to demonstrate the targeted sites in proteins. The CD cleavage sites in some native proteins, such as bovine serum albumin (BSA) [22], hemoglobin [26], [27], actin [28], antichymotrypsin and kallistatin [29], were therefore examined individually and hydrophobic amino acids potentially associated with the scissile bonds were proposed as targeted sites. Moreover, it was noted that CD preferred sites involved in the α-helical conformation of myoglobin and cytochrome c [22]. Recently, proteomic approaches have emerged as a powerful tool for screening the protein substrates and characterizing the features of proteases [30], [31]. Global profiles have indicated that CD proteolysis mainly occurs between hydrophobic residues, with a strong preference for leucine and phenylalanine [27], [32], [33]. Additionally, the cleavage activity of CD in target proteins not only relies on the linear sequences of amino acids but is also decided, at least in part, by the protein’s spatial structure [34]. Nevertheless, little research has focused on the overall evaluation of CD cleavage efficiency in typically targeted proteins, which has prevented a detailed analysis of the cleavage characteristics of CD and has led to confusion regarding which elements are critically important for CD cleavage.

In the present study, we sought to address an effective method to systematically inspect the cleavage sites targeted by human CD in native proteins. Using liquid chromatography-tandem mass spectrometry (LC-MS/MS) and by searching for non-tryptic peptides, the database of CD-cleaved peptides was generated, which was beneficial for further exploring the specificity of the CD cleavage sites. Moreover, we identified more proteins that were resistant to CD cleavage in a CD-knockdown cell line via two-dimensional gel electrophoresis (2DE) and MS. With respect to the proteins that were sensitive or insensitive to CD cleavage and the peptides generated by CD, we provide convincing evidence and a reasonable hypothesis regarding the CD-related characteristics of cleaved peptides and CD accessibility to the target proteins.

## Materials and Methods

### Reagents

Human CD, human apo-transferrin (TF), human serum albumin (HSA), BSA, porcine serum albumin (PSA), porcine hemoglobin (HB), chicken OVA and microbial pepstatin A were purchased from Sigma-Aldrich (St. Louis, MO, USA). Trypsin was obtained from Promega (Madison, WI, USA), chymotrypsin from Roche (Indianapolis, IN, USA) and Lipofectamine 2000 and geneticin from Invitrogen (Carlsbad, CA, USA). Immobiline pH gradient (IPG) DryStrips (pH 3-10 NL, length 18 cm) and the corresponding buffer were obtained from GE Healthcare (Piscataway, NJ, USA). Recombinant human aldo-keto reductases (AKRs) and mouse glutathione S-transferases (GSTs) were generated in our laboratory using an *E. coli* expression system.

### Proteins Cleaved by CD

In order to assess the favorable conditions of CD cleavage, the proteins that are sensitive or insensitive to CD cleavage like BSA and OVA, were incubated with CD under different digestion conditions, such as buffer pHs, reaction temperatures and incubation time. After evaluating all the conditions of CD cleavage, especially considering to detect the amino acid sites more susceptible to CD cleavage, optimal reaction conditions were selected in which cleavage targets at a final concentration of 1.5 µM were incubated with 0.2 U CD in 100 mM sodium citrate buffer (pH 3.5) for 3 h at 37°C. To estimate the efficiency of CD cleavage, the mixtures of the targets and CD before and after incubation were loaded onto 12% SDS-PAGE gels, followed by silver nitrate staining.

### Identification of the Peptides Generated from CD Cleavage by MS

The peptides generated from CD cleavage were diluted with 0.1% formic acid, separated using Easy-nLC (Bruker Daltonics, Karlsruhe, Germany), mounted with a C18 column (75 µm×150 mm, LC Packings) and run with a linear elution gradient of 5–35% acetonitrile at flow rate of 400 nL/min. The separated peptides were delivered directly into a high-capacity amaZon ETD MS ion trap spectrometer (Bruker Daltonics) using nanoESI spray. The MS/MS spectra of the peptide fragments were converted to mgf files using DataAnalysis 3.4 (Bruker Daltonics), delivered to the peptide search engine Mascot 2.3 (Matrix Science, Boston, MA, USA) and searched against the Swiss-Prot database 57.15 with the following search parameters, enzyme: None, peptide mass tolerance: 0.6 Da, fragment mass tolerance: 1.0 Da and instrument: ESI-TRAP. Considering the limited MS/MS spectra generated from the selected target proteins, a consecutive approach was adopted to reduce the false discovery rate (FDR) in the identified peptides. First of all, the MS/MS spectra were searched with Mascot against Swiss-Prot database and the database generated from all the target substrates in parallel. A truly positive peptide was defined to locate at the rank one of the search results against Swiss-Prot and reach the confidence level above 95% in the search against the substrate database. Then the spectra corresponding to the identified peptides were manually examined.

### Denaturation and Electroelution of OVA

OVA was denatured in sample buffer containing 2% SDS and further separated via SDS-PAGE. The corresponding band at approximately 41 kDa was excised, soaked in electroelution buffer (25 mM Tris–HCl, 192 mM glycine and 0.5% SDS, pH 8.3) and placed into a dialysis bag with a cut-off of 3,500 Da (Solarbio, Beijing, China). Electroelution was carried out for 2 h at 100 mA in the same buffer in a horizontal electrophoretic device. To concentrate the protein and remove SDS, the eluted protein was loaded onto an Amicon ultra filter (Millipore, Billerica, MA, USA) and centrifuged at 10,000 g.

### Generation of a Stable CD-knockdown Cell Line

The shRNA oligonucleotides targeting to CD mRNA were designed according to Ohri [35], with the following sequences: 5′ gatccggcaaaggctacaagctgtttcaagagaacagcttgtagcctttgccttttttggaaa 3′ and 5′ agcttttccaaaaaaggcaaaggctacaagctgttctcttgaaacagcttgtagcctttgccg 3′. The oligonucleotides were synthesized in Sangon (Beijing, China), linearized using *Bam*H I and *Hin*d III, and inserted into pSilencer 3.0-H1 (Ambion, Austin, TX, USA) to construct the transfection vector pSilencer 3.0-CD. A549 cells were obtained from the Chinese Academy of Medical Sciences and Peking Union Medical College, China. A549 cells were cultured in RPMI-1640 medium supplemented with 10% heat-inactivated FCS (Sigma-Aldrich) at 37°C under 5% CO_2_. The cells were transfected with pSilencer 3.0-CD and empty vector using Lipofectamine 2000. The transfected colonies were picked 2 weeks after transfection in the presence of geneticin and were subsequently expanded into cell lines, designated A549-CR and A549-EV.

### Protein Extraction from A549-CR Cells

A549-CR cells were harvested with a rubber policeman in chilled PBS with 1 mM PMSF and 2 mM EDTA and were ruptured via repeated passages through a 26-gauge needle. The lysate was centrifuged at 20,000 g at 4°C for 30 min. The supernatant as the cytosolic fraction were further diluted with 100 mM sodium citrate, pH 5.0, followed by centrifugation at 20,000 g to clear off the precipitates. The retained supernatants were incubated with/without 1.2 U CD at 37°C overnight.

### Evaluation of the Efficiency of CD Cleavage of the A549-CR proteins using 2DE and MS

The A549-CR proteins treated with/without CD were mixed with a rehydration solution containing 8 M urea, 4% (w/v) CHAPS, 20 mM DTT and 0.5% IPG buffer and then applied to IPG strips. After rehydration, isoelectrofocusing was carried out at 56 kVh using IPGphor (GE Healthcare) at 20°C. The focused strips were equilibrated in a solution(6 M urea, 50 mM Tris-HCl, 30% glycerol, 2% SDS and bromophenol blue) that contained 1% DTT (v/v) during the first equilibration step and 2.5% iodoacetamide (v/v) during the second equilibration step (15 min per equilibration step) and then transferred to 13% SDS-PAGE gels in Ettan DALT II (GE). The protein spots were visualized via silver nitrate staining, and 2DE images were acquired with ImageScanner (Amersham Biosciences, Piscataway, NJ, USA). Image analysis was performed with Imagemaster 5.0 (GE Healthcare). The 2DE spots showing change ratios of less than 1.2-fold between the samples with/without CD treatment were considered to represent CD-resistant spots. These spots were excised from gels, reduced, alkylated and digested overnight with trypsin. The digested products were identified by UltraFlex II MALDI-TOF-TOF MS (Bruker Daltonics). The obtained MS and MS/MS spectra were processed using FlexAnalysis 3.0 and BioTools 3.0 (Bruker Daltonics). Protein searches were performed with Mascot 2.3 against the Swiss-Prot database 57.15, with the following parameter settings: monoisotopic mass accuracy <100 ppm; 1 missed cleavage; carbamidomethylation of cysteine as a fixed modification and oxidation of methionine, N-terminal pyroglutamylation (peptide) and N-terminal acetylation (protein) as variable modifications.

## Results

### CD Cleavage Characteristics of BSA

**Figure 1:**
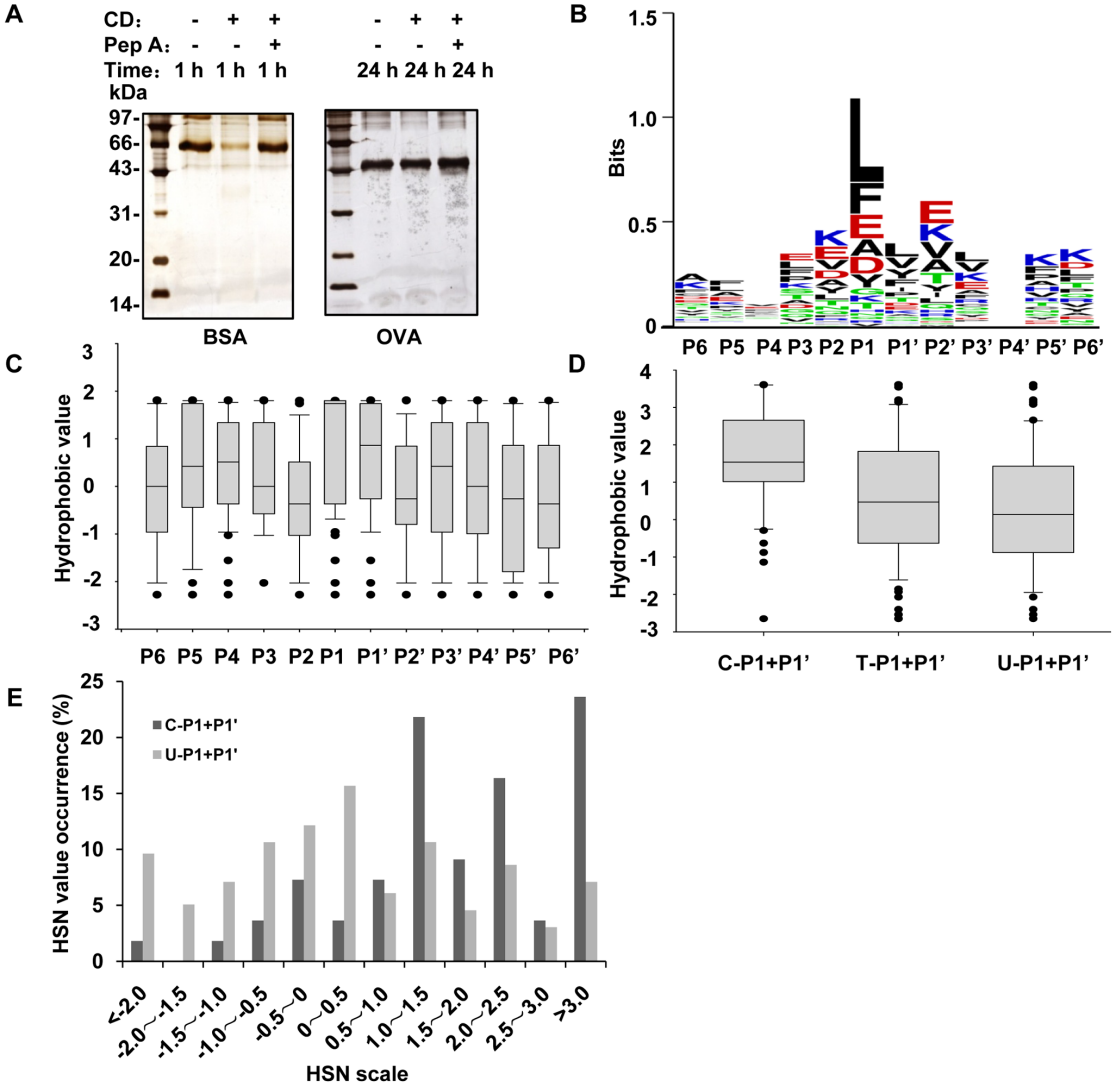
The cleavage efficiency and cleavage characteristics of CD in BSA. *A*, The sensitivity of BSA and OVA to CD cleavage was examined via SDS-PAGE after the two proteins were incubated with/without CD and pepstatin A. *B*, The CD specificity profile for BSA is depicted using sequence logos. Six amino acids are represented as P6…P1 and P1’…P6’, which are located at N- and C-termini near the scissile site, respectively. The sequence logo ordinate is scaled in bits based on Schneider method [36]. Color coding: acidic residues in red, basic residues in blue, polar residues in green and hydrophobic residues in black. *C*, The hydrophobicity distributions of all the residues at 12 positions around the termini of the CD-cleaved BSA peptides were estimated with box plots. *D*, Comparison of the median values of T-P1+P1’, C-P1+P1’ and U-P1+P1’ in BSA. The analysis was performed for the six amino acids whose occurrence rates at P1 received the top ranks. *E*, Comparison of the HSN distributions between the cleaved and undetected peptides from BSA. The HSN values were broadly divided into 12 intervals.

To characterize the features associated with CD cleavage in proteins, we selected two typical proteins, BSA and OVA, that were previously reported to be sensitive or insensitive to CD, and globally identified their cleavage sites using MS. We first used these two proteins to determine the CD cleavage activity in citrate buffers with pH levels ranging from 3.0 to 7.0 (data not shown). In accordance with previous observations, we found that CD exhibited the highest BSA cleavage activity at pH 3.5. However, CD failed to cleave OVA in the tested pH range. Thus, we set pH 3.5 as the optimal CD reaction conditions for the following experiments. The efficiency of CD cleavage of BSA and OVA was also evaluated. As illustrated in Figure 1A, BSA was quite sensitive to CD cleavage, and the BSA band nearly disappeared within 1 h of incubation, whereas the OVA band intensity remained constant, even after prolonged incubation for up to 24 h. Moreover, the cleavage of BSA by CD was significantly inhibited by pepstatin A, an inhibitor of CD, indicating that the disappearance of the BSA band was due to CD proteolysis rather than random hydrolysis.

There is no parameter selection for CD digestion in protein search engines such as Mascot and SEQUEST. Therefore, setting no-enzyme in Mascot was used to search for peptides generated by CD based on MS/MS signals. To ensure the feasibility and accuracy of this search approach, we digested BSA with trypsin, performed LC-MS/MS and then searched the peptides using Mascot with the enzyme parameter set as “none” rather than “trypsin”. The search results revealed that all of the qualified peptides were well matched with the characteristics of tryptic cleavage; *i.e.*, all of the C-termini contained arginine or lysine (Table S1). We therefore adopted the same approach to search the BSA peptides elicited from CD cleavage. A total of 64 peptides were identified, including 67 cleavage sites. As shown in Table S2, no specifically oriented residue(s) were observed in the termini of these peptides, with 14 different residues detected at the C-termini and 17 different residues detected at the N-termini. Sequence logo was adopted to discern the distribution patterns of the amino acids near the cleaved termini [31], [36]. A sequence logo consists of a stack of amino acid symbols at each position. The total height of the stack indicates the sequence conversation at that position, while the size of each symbol within the stack indicates its relative frequency at that position. As shown in Figure 1B, the logos deliver the following information: 1) there are no conservative motifs among the BSA peptides; 2) there is no obvious preference for amino acid residues at positions other than P1; 3) several residues are much more preferred by CD at P1, such as L, F, E, A and D, which are all hydrophobic amino acids (the acidic amino acids become a little hydrophobic at pH 3.5), and 4) although P1 is dominantly occupied by hydrophobic residues with a total occurrence frequency nearly 83%, 7 kinds of hydrophilic amino acids appear at P1 with a total of 17% cleavage preference. The occurrence frequencies of the residues at each position in the logos are listed in Table S3. These results are generally in agreement with previous observations that the hydrophobic residues are favored by CD. The question is why CD also favors to chop these hydrophilic residues at P1. We then specifically checked the residues at P1’ corresponding to P1 sites with hydrophilic residues and found that most of the P1’ sites were hydrophobic residues (10 out of 11). We therefore propose that CD not only favors hydrophobic residues at P1 but also prefers the hydrophobic microenvironment contributed by neighboring residues at P1’. The median hydrophobic scores of all the residues at each position in the logos (Fig. 1B) were estimated according to the amino acid hydrophobicity determined by Cowan method [37], and these are illustrated in Figure 1C. Compared with the other positions, the median hydrophobic scores at P1 and P1’ were dramatically higher, implying that P1’ is important in CD recognition.

Considering that CD cleavage is likely correlated with the hydrophobic microenvironment contributed by P1 and P1’, we introduce a new concept to describe the hydrophobicity of neighboring residues referred to as the hydrophobic scores of neighbors (HSN), where an HSN value represents the sum of the hydrophobic scores of two neighboring residues. We analyzed the CD cleavage characteristic of BSA in term of HSN. As approximately 81% of the P1 positions in the CD-cleaved BSA peptides were occupied by the 6 residues: L, F, E, A, D and Y, we extracted all the P1/P1’ residue pairs in BSA sequence that consist of all the 6 residues (as P1) and their corresponding neighbor residues (as P1’), and broadly divided these pairs into 3 groups: all of the P1/P1’ pairs in BSA sequence (T); all of the pairs detected via LC-MS/MS (C); and all of the pairs undetected (U). The HSN value for a P1/P1’ pair is the sum of the hydrophobic sores of the residue at P1 and its neighboring residue at P1’, which was calculated according to Cowan method [37]. Figure 1D presents the median HSN values from each group and demonstrates that the median value in the C group is significantly higher than those of the other two groups. Moreover, the HSN occurrence frequency, denoted as the ratio of the number of the P1/P1’ pairs with certain HSNs to the total pair number, was plotted against the scale of the HSN intervals to determine which HSN value could be regarded as the threshold for CD cleavage of BSA. The data presented in Figure 1E demonstrate that the HSN interval of 0.5–1.0 serves as a cutoff, with all of the HSN occurrence frequencies in the C group higher than U falling on the right, while those in the U group greater than C fall on the left. Our results therefore suggest that an HSN value of 0.5–1.0 in BSA is an indicator of CD cleavage.

### CD Cleavage Characteristics of OVA

**Figure 2:**
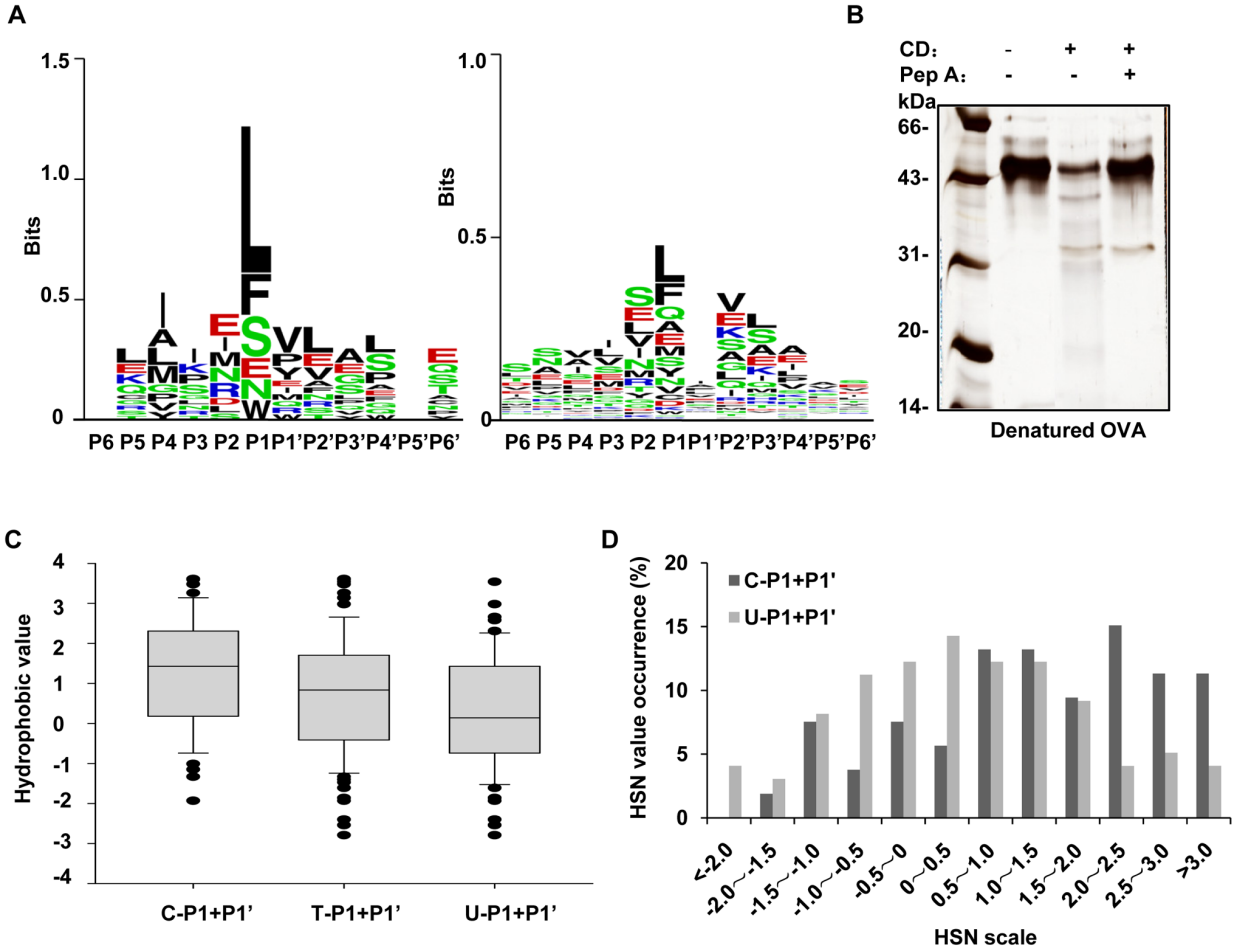
Analysis of CD cleavage characteristics in denatured OVA. *A*, Sequence logos of the CD cleavage sites in native OVA (*left*) and denatured OVA (*right*). *B*, The sensitivity of denatured OVA to CD cleavage was examined via SDS-PAGE, in which the denatured protein was incubated with/without CD and pepstatin A for 3 h. *C*, The median T-HSNs, C-HSNs and U-HSNs in denatured OVA are depicted using box plots. *D*, Comparison of the HSN distributions between the cleaved and undetected peptides from denatured OVA.

Although no digestion of OVA was visualized via SDS-PAGE with silver staining after CD cleavage for 24 h, a few OVA peptides cleaved by CD could be detected via LC-MS/MS. A total of 14 cleavage sites were identified and mapped in the form of sequence logos (Fig. 2A-left). Similar to BSA, L and F remain at the top in the P1 position. The occurrence frequencies of each residue at 12 positions are listed in Table S4. Whether does the spatial structure of OVA prevent CD access to it? We denatured OVA and tested the action of CD on the denatured form. After SDS denaturation and electroelution, OVA became sensitive to CD cleavage; specifically, OVA was cleaved by CD completely within 3 h and was protected against CD cleavage by the addition of pepstatin A (Fig. 2B). We identified 87 OVA peptides generated by CD via LC-MS/MS, including 85 cleavage sites. The sequence logos for the sequences at the termini of OVA peptides are presented in Figure 2A-right and the occurrence frequencies of each residue at different positions are listed in Table S5. As shown in Figure 2A-right, the residues L and F are ranked as the top cleavage sites, while the total percentage of hydrophobic amino acids at P1 is 74%, suggesting that after CD accesses the denatured OVA cleavage regions, it still favors cleavage at hydrophobic residues. We then utilized the HSN to examine the CD cleavage characteristics of OVA. By selecting the P1/P1’ pairs consisted of the top 6 residues appearing at P1 in Figure 2A-right (as P1) and their corresponding residues (as P1’) in OVA sequence, the HSNs were calculated and the median HSNs were evaluated in the same 3 groups described above. The median HSN value in group C is clearly higher than those for groups T and U (Fig. 2C). The HSN occurrence frequencies were analyzed against the HSN interval scale. The result reveals that the HSN interval of 0.5–1.0 is also a clear indicator of CD cleavage in OVA (Fig. 2D).

### Analysis of the General Cleavage Features of CD

As the similarities of the CD cleavage characteristics of BSA and denatured OVA were clearly identified, whether these cleavage characteristics could be generalized for other proteins is still questionable. Thus, we initiated a systematic survey towards the CD cleavage sites in several proteins. We selected 3 protein groups: 1) proteins commonly used as CD substrates, such as TF and HB; 2) protein family members sharing similar protein structures and varying amino acid sequences, such as the albumin (BSA, HSA and PSA), AKR (AKR1A1, AKR1B10, AKR1C1 and AKR1C3) and GST (GST-A3, GST-M1 and GST-P1) families; and 3) proteins that experience autolysis, such as CD, trypsin and chymotrypsin. All of the proteins in the 3 groups were incubated with CD, followed by SDS-PAGE. The results indicated that the proteins in the former 2 groups were all sensitive to CD, and then the CD-cleaved peptides of these proteins were detected via LC-MS/MS. After searching against Swiss-Prot database, 556 peptides were found at rank one. Further peptide search against the substrate database and manual spectrum check resulted in the removal of 36 peptide candidates with lower confident scores. A total of 520 CD-cleaved peptides (569 cleavage sites) generated from these proteins were finally identified, including 34 CD peptides due to CD self-cleavage. The CD-cleaved peptide database is referred to as the CCPD, which is presented in Table S2. The 569 cleavage sites are presented in Table S6. The number of the cleavage sites in each protein and the occurrence frequencies of 6 residues at P1, which showed the highest detection rates at P1 in the CCPD, are illustrated in Table 1.

**Table 1:** The CD cleavage site numbers and the occurrence frequencies of the top 6 amino acids at P1 in each protein.

Protein	Site No.	Amino acid occurrence (%)						
		L	F	A	Y	E	D	Sum
1.BSA	67	36	13	9	6	10	7	81
2.HSA	55	25	27	7	5	13	7	84
3.PSA	75	32	13	8	4	9	4	70
4.TF	53	28	11	17	9	8	9	82
5.HB	49	23	12	12	2	2	12	63
6.AKR1A1	26	54	8	8	4	0	4	78
7.AKR1B10	60	22	15	12	2	10	7	68
8.AKR1C1	15	20	20	7	13	13	0	73
9.AKR1C3	22	36	18	0	5	9	9	77
10.GSTs	22	36	18	0	9	5	9	77
11.D-OVA	85	21	13	7	6	7	4	58
12.CD	40	23	18	5	18	8	3	75

The numbers of CD cleavage sites in 12 proteins, the occurrence frequencies of the top 6 residues (L, F, A, Y, E and D) appearing at P1 in the CCPD and the total occurrence frequencies of the 6 residues in each protein are listed below.

BSA, HSA and PSA belong to the same family, presenting α-helical structures and showing over 60% homology. AKR proteins share an α/β barrel structure and approximately 40% homology. Through analysis of the CD-cleaved protein families, we found approximately 30% of the cleavage sites in BSA, HSA and PSA overlapped; approximately 30% of the cleavage sites were identical between AKR1A1 and AKR1B10, while approximately 49% were identical between AKR1C1 and AKR1C3. These data demonstrated that several peptides with identical amino acid sequences in all of the members of a protein family were highly sensitive to CD cleavage. Therefore, CD appears to possess a recognition ability oriented toward certain peptide sequences. On the other hand, the proteases responsible for autolysis examined in this study, *i.e.*, trypsin, chymotrypsin and CD, did not show obvious changes in their molecular masses after incubated with CD.

**Figure 3:**
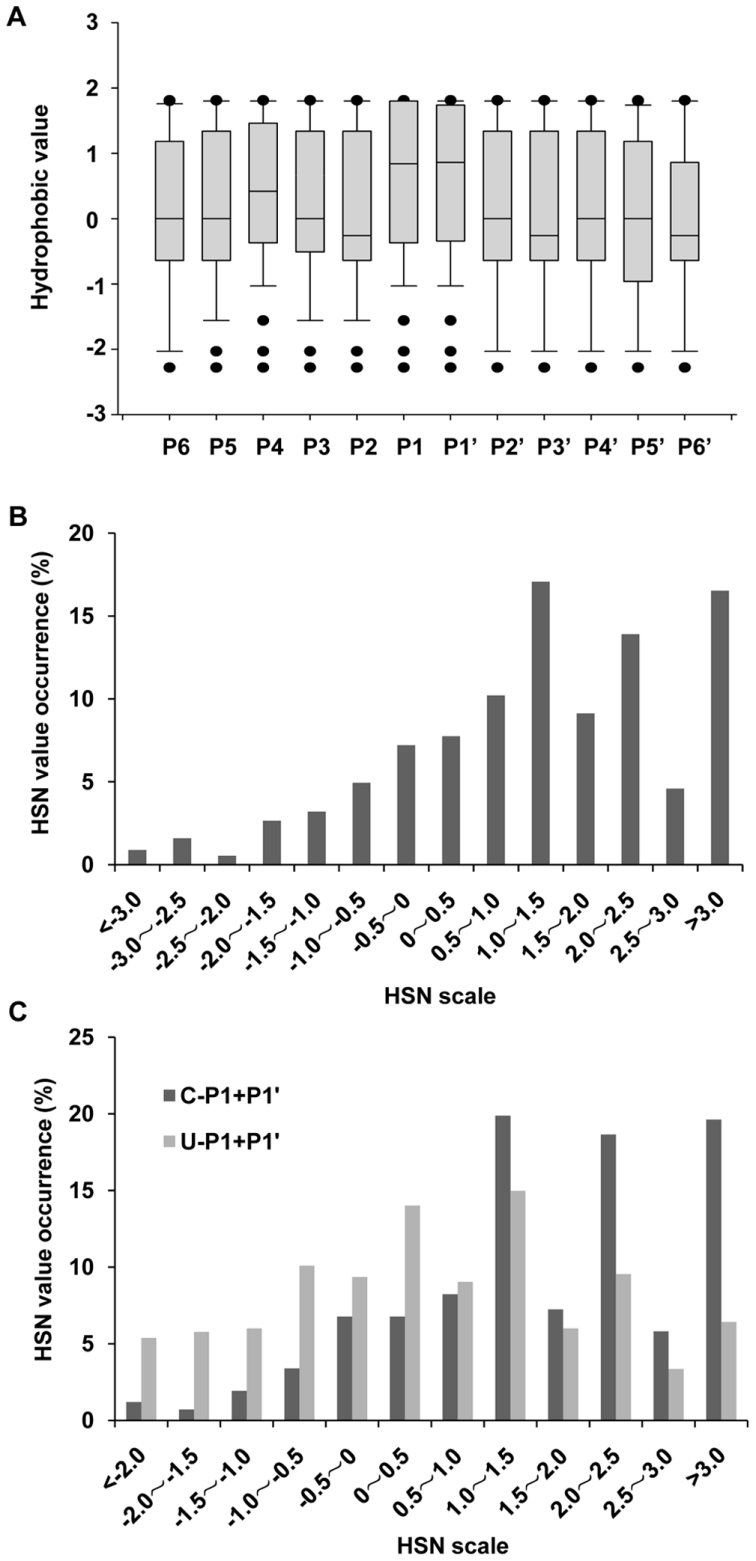
Analysis of hydrophobicity distributions in the CCPD. *A*, The hydrophobicity distributions of all the residues at 12 positions around the termini of the peptides in the CCPD were estimated with box plots. *B*, The HSN profile in the CCPD. The occurrence frequencies of all the HSNs in the CCPD were plotted against the HSN axis, which was divided into 14 intervals. *C*, Comparison of the HSN distributions between the cleaved and undetected peptides in the CCPD. The HSN values were estimated for the top 6 amino acids.

The plentiful information in the CCPD offers an opportunity to evaluate the overall characteristics of the CD cleavage of protein substrates. Similar to Figure 1C, the median hydrophobic scores of the residues at 12 positions around the scissile sites in the CCPD are illustrated in Figure 3A. Even with the CD-cleaved peptides over 500, the conclusion drawn from Figure 3A is in a good agreement with that from Figure 1C, in which the median of hydrophobicity at either P1 or P1’ is obviously higher than that at other positions. Thus, the hydrophobic microenvironment of CD recognition sites is not only applied to BSA or OVA, but also suitable to many proteins that are easily cleaved by CD. We calculated the HSNs of all the P1/P1’ pairs in the CCPD and plotted these together with the occurrence frequencies against the HSNs. As shown in Figure 3B, when the HSNs ranged from −3.0–1.5, higher HSN values for the P1/P1’ pairs are associated with more peptides generated by CD cleavage. However, when the HSNs ranged from 1.5–3.0, the occurrence frequencies appear to not always be HSN dependent, implying that there is an HSN threshold for CD cleavage and that once the threshold is reached, the HSN parameter may not be sensitive to such proteolysis. Since the top 6 residues showing the highest appearance rates at P1 in the CCPD are L, F, E, A, D and Y, we adopted the same strategy as described in BSA section to compare the occurrence frequencies of C-HSN and U-HSN. The results are in agreement with the conclusion arising from Figure 1E that the HSN interval of 0.5–1.0 is likely to be a cutoff to evaluate susceptibility to CD cleavage (Fig. 3C).

The HSN values above were calculated using Cowan method [37]. Then we recalculated and compared the C-HSN and U-HSN in the CCPD using Wimley method [38]. The results presented in Figure S1 provide solid support for Cowan method [37], even when the hydrophobic scores calculated from the two methods are slightly different, the deductions based on hydrophobic prediction are quite close. Thus, we are confident that the HSNs of peptides are indicative of their susceptibility to CD cleavage.

### Why are Some Native Proteins Insensitive to CD Cleavage?

**Figure 4:**
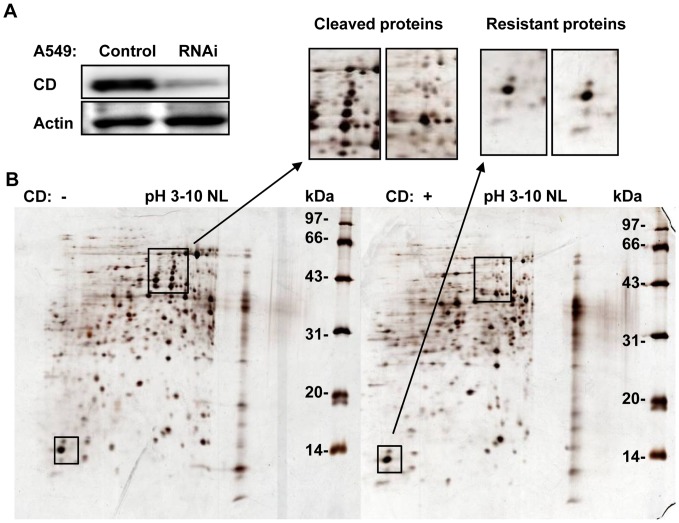
Screening of proteins that were insensitive to CD cleavage in A549-CR cells. *A*, The expression of CD in A549 cells was evaluated by Western blotting. The control was transfected with empty vector. *B, C*omparison of the 2DE images obtained from lysates of A549-CR cells treated with/without CD. The insets represent the typical 2DE images showing spots that were sensitive or insensitive to CD cleavage.

As mentioned above that some native proteins, such as OVA, trypsin, chymotrypsin and CD itself were insensitive to CD cleavage, the limited cases could not deliver enough information to explain such insensitivity. We further sought more native proteins that are generally insensitive to CD cleavage in cells via 2DE. To prevent endogenous CD cleavage of proteins, a stable CD-knockdown cell line, A549-CR, was generated using an RNAi approach (Fig. 4A). Considering that the conditions at pH 3.5 might cause global protein hydrolysis, we carefully evaluated the reaction conditions that allowed the proteolytic functions of CD and minimized other protein hydrolysis. We found the use of 100 mM sodium citrate, pH 5.0, to be feasible for the experimental purpose. Thus, A549-CR lysates were incubated with/without CD in this buffer for 12 h, followed by 2DE. As shown in Figure 4B, most of the spots in the CD+ image exhibited dramatic changes, either in terms of the spot positions or intensities, as compared to the CD- treatment, indicating that CD showed a proteolytic function for most proteins. A total of 342 spots were detected in the CD- 2DE image; of these, 175 only appeared in this sample, while 167 were shared with the CD+ sample but presented relatively higher spot intensities. Additionally, a total of 322 spots were perceived in the CD+2DE image, 155 of which were only found in this sample. These results revealed that most of the native proteins in the cells were sensitive to CD cleavage, even at pH 5.0. On the other hand, 19 spots did not show any significant changes in their spot intensities. Of these spots, we identified 4 unique proteins by MALDI TOF/TOF MS: nucleoside diphosphate kinase A (NDKA), thioredoxin (Trx), fatty acid-binding protein (epidermal, FABP5) and coactosin-like protein (COTL1).

Thus, a total of 8 proteins, including OVA, trypsin, chymotrypsin, NDKA, Trx, FABP5, COTL1 and CD itself, were found insensitive to CD. To explore the causal factors related to such resistance, we attempted to trace three biochemical parameters in these proteins. First, as HSN is a critical parameter for judging the likelihood of CD-digested sites, we asked whether these proteins possess generally lower HSNs, which would generate a weakly hydrophobic environment unfavorable to CD cleavage. We evaluated the HSNs of the 8 proteins and obtained the median HSN values listed in Table S7. As compared to the median HSNs of the proteins that were sensitive to CD cleavage, the median values for the proteins that were insensitive to CD cleavage does not display significant differences, with the exception of NDKA and FABP5, implying that HSN is not an appropriate parameter for distinguishing whether intact forms of proteins are sensitive/insensitive to CD cleavage. Second, according to van Noort and van der Drift’s view [22], CD cleavage is favored by an α-helical structure. Thus, it is possible that these proteins are resistant to CD cleavage because they are rich in β-sheets with less α-helical structure. According to SOPMA secondary structure prediction and PDB database, we were able to obtain information on the secondary structure distributions and spatial structures of 18 proteins through theoretical analysis and experimental examination; the results included 8 resistant proteins identified in the current study (OVA, NDKA, COTL1, Trx, FABP5, trypsin, chymotrypsin and CD), 5 substrates used in the current study (HSA, HB, TF, AKR1B10 and GST-P1) and 5 substrates reported previously (actin, neuroleukin, profilin-1, cytochrome c and annexin I) [33]. This information is listed in Table 2. Generally, all of the proteins in the group sensitive to CD cleavage contain a relatively higher ratio of α-helices, whereas the proteins that are insensitive to CD cleavage exhibit a relatively higher ratio of β-sheets. This suggests that α-helical structures in intact proteins are relatively accessible to CD. Importantly, the CD cleavage sites in a protein appear not to be α-helix- or β-sheet-dependent because several CD cleavage sites, such as those in AKRs and OVA, were located in β-sheets. Third, we examined whether common spatial structures are shared by proteins that are sensitive/insensitive to CD cleavage. According to the SCOP principle, X-ray structures can be broadly categorized into 5 classes: all α-helices (α), all β-sheets (β), parallel β-sheets (α/β), antiparallel β-sheets (α+β) and mixed α/β and α+β (αβ) structures. As shown in Table 2, among the proteins that were insensitive to CD cleavage, trypsin, chymotrypsin, FABP5 and CD belong to the β class, NDKA and COTL1 to the α+β class, Trx to the α/β class and OVA to the αβ class; in contrast, the proteins that were sensitive to CD cleavage all belong to the α, α/β or α+β class. This classification indicates that an intact protein with an all α-helical structure is favorable to CD cleavage, whereas proteins with an all β-sheet structure appear to conclusively be CD-resistant. Together, our results led us to conclude that the insensitivity to CD cleavage among the 8 identified proteins is likely a result of their intact, β-sheet-rich structure.

**Table 2:** The secondary structure (α-helix and β-sheet) constitution of proteins that were resistant/sensitive to CD.

Protein		PDB		SOPMA		SCOP Classification
		α-helix	β-sheet	α-helix	β-sheet	
		(%)	(%)	(%)	(%)	
Resistant protein						
OVA		30	32	44	15	Multi-domain proteins (α and β)
NDKA		46	17	45	19	α and β proteins (α+β)
COTL1		33	29	46	20	α and β proteins (α+β)
Trx		30	27	44	17	α and β proteins (α/β)
FABP5		15	56	31	35	All β proteins
Trypsin		10	34	17	29	All β proteins
Chymotrypsin	Chain B	2	38	8	30	All β proteins
	Chain C	25	37	26	19	All β proteins
CD	Chain A	7	54	9	36	All β proteins
	Chain B	19	41	13	38	All β proteins
Sensitive protein						
HSA		70	0	69	3	All α proteins
HB	Chain A	75	0	61	6	All α proteins
	Chain B	77	0	58	11	All α proteins
TF		31	18	31	19	α and β proteins (α/β)
AKR1B10		36	16	36	18	α and β proteins (α/β)
GST-P1		58	8	48	11	All α and α/β (C−/N-terminal)
Actin		40	19	32	22	α and β proteins (α/β)
Neuroleukin		53	12	50	13	α and β proteins (α/β)
Profilin-1		26	18	23	34	α and β proteins (α+β)
Cytochrome c		40	1	36	11	All α proteins
Annexin I		64	0	71	2	All α proteins

The columns from left to right contain the protein name, the percentage of α-helices and β-sheets in the PDB database, the percentage of α-helices and β-sheets predicted by SOPMA and the classification in SCOP.

## Discussion

A proteomic approach is generally accepted for the investigation of protease catalytic characteristics. We employed such an approach to monitor CD cleavage products and generated a database, the CCPD, which provides a solid foundation for the extraction of common biochemical features of the CD-cleaved peptides. Our data confirmed that CD is not a protease that can specifically recognize and cleave a specific motif or amino acid residue. In the CCPD, many residues appeared at P1, especially residues L, F, A, Y, E and D showing much higher occurrence frequencies than the other residues. It is noticeable that CD preferred two hydrophilic residues (E and D) at P1. In an acidic environment, these two acidic amino acids are highly protonated, which results in augmentation of their hydrophobicity. According to Cowan method [37], the hydrophobic scores for residues E and D could increase from −1.95 and −2.15 at pH 7.5 to −0.37 and −0.51 at pH 3.0. Under the examined CD digestion conditions, these two residues should be regarded as hydrophobic residues. These results hence are generally in agreement with previous observations that CD preferred to cleave hydrophobic residues at P1 [22], [27], [32]. However, other data appear to contest this notion. For instance, a number of hydrophilic residues are located at P1 in the CCPD. So the proteolytic characteristics of CD cannot simply be described as being dependent on the hydrophobicity of amino acids at P1. On the basis of analysis of the amino acid hydrophobicity around the cleavage sites, we found that CD tended to hydrolyze the peptide bonds containing at least one hydrophobic residue. The hydrophobic microenvironment contributed by residues at P1 and P1’ is a preferential factor for CD recognition. We further proposed the HSN as a new parameter for evaluating the CD-cleaved sites in target proteins and showed that an HSN threshold of 0.5–1.0 could be used to judge the CD cleavage efficiency for target peptides. Furthermore, our hypothesis is able to explain previous observations regarding CD cleavage. For instance, the recently reported CD-cleaved sites identified in caspase-8 [13], Bid [39] and Aven [40] contain hydrophobic residues at either P1 or P1’ and are associated with an HSN of more than 1.0. We also carefully examined the sites of CD cleavage reported by Impens *et al.* [33]. Approximately 70% of the 584 cleavage sites corresponded to L and F located at P1, whereas hydrophobic amino acids consistently appeared at the P1’ sites corresponding to the hydrophilic residues at P1. Estimation of the HSNs for all of the 584 sites indicated that almost 90% exhibited HSN values above the 0.5–1.0 threshold. Hence, the HSN principle appears to be generally suitable for all CD substrates, at least for the linear sequences of peptides.

Although the current study mainly focused on the characteristics of CD cleavage, it is possible that the HSN principle could also be applicable to other aspartyl proteases. We briefly examined the HSNs of the cleavage sites of some aspartyl proteases, including pepsin, HIV-1 protease and chymosin. Over 70% of 1,344 pepsin cleavage sites [41] presented HSNs ≥0.5; approximately 80% of 148 HIV-1 protease cleavage sites [42] exhibited HSN values equal to or above the threshold of 0.5–1.0; and nearly 58% of 48 chymosin cleavage sites [43] exhibited HSNs over the threshold of 0.5–1.0. These three aspartyl proteases all preferred hydrophobic amino acids at P1, such as L and F. Although these cleavage sites presented limited data with respect to the overall evaluation of the proteolytic characteristics of these aspartyl proteases, the findings provide two interesting facts related to CD cleavage: 1) L and/or F always occupy the sites associated with the top cleavage preference and 2) the HSN values at the majority of cleavage sites are over the threshold of 0.5–1.0. This observation indicates that the aspartyl proteases are likely to share a similar mechanism for the cleavage of their target proteins.

Our data revealed that native OVA was hardly cleaved by CD, while denatured OVA was quite sensitive to CD cleavage, indicating that the denaturation broke the structural hinge of OVA and made the cleavable regions accessible to CD. The question is which spatial factor affects CD access to its target protein. We checked the structures of some proteins in SCOP and found that proteins with all β-sheets are insensitive to CD cleavage, like trypsin and FABP5; and proteins with all α-helices are sensitive to CD cleavage, like HSA and HB (Table 2). However, with regard to the proteins with mixed secondary structures such as α+β or α/β, their susceptibility to CD cleavage seems not to follow a clear regulation. For instance, OVA and profilin-1 possess relatively similar distributions of α-helix and β-sheet no matter in PDB or SOPMA (Table 2); however, they display so different susceptibility to CD cleavage. If protein secondary structure does play a key role in CD access, the coil structure between α-helix and β-sheet is likely a considerable factor to regulate such susceptibility. On the basis of the currently available data, we could not draw a conclusion that how to set up a structural threshold for CD access to a protein. On the other hand, two deductions are acceptable, 1) a protein with all α-helices is sensitive to CD cleavage, whereas a protein with all β-sheets is insensitive to CD; and 2) once the spatial structure of a protein insensitive to CD cleavage is cracked, CD can attack its scissile sites with high HSN values.

## Supporting Information

Figure S1 Comparison of the HSN distributions between the cleaved and undetected peptides in the CCPD. The hydrophobic values of amino acids were analyzed according to Wimley method [38].
(TIF)

Table S1 The tryptic BSA peptides identified via LC-MS/MS and no enzyme search in Mascot.
(DOC)

Table S2 CD-cleaved peptide database (CCPD).
(DOC)

Table S3 The corresponding occurrence frequencies of the residues at each position in Figure 1B.
(DOC)

Table S4 The corresponding occurrence frequencies of the residues at each position in Figure 2A-left.
(DOC)

Table S5 The corresponding occurrence frequencies of the residues at each position in Figure 2A-right.
(DOC)

Table S6 List of human CD cleavage sites in the substrate proteins used in this study.
(DOC)

Table S7 Comparison of the T-HSNs among the substrates used in this study (*upper panel*) and resistant proteins verified in this study (*lower panel*).
(DOC)
