# Supplementary figures and images for: Proteolytic Characteristics of Cathepsin D Related to the Recognition and Cleavage of Its Target Proteins
Source: PLoS One. 2013 Jun 20;8(6):e65733. doi: 10.1371/journal.pone.0065733 (PMC3688724; doi:10.1371/journal.pone.0065733)

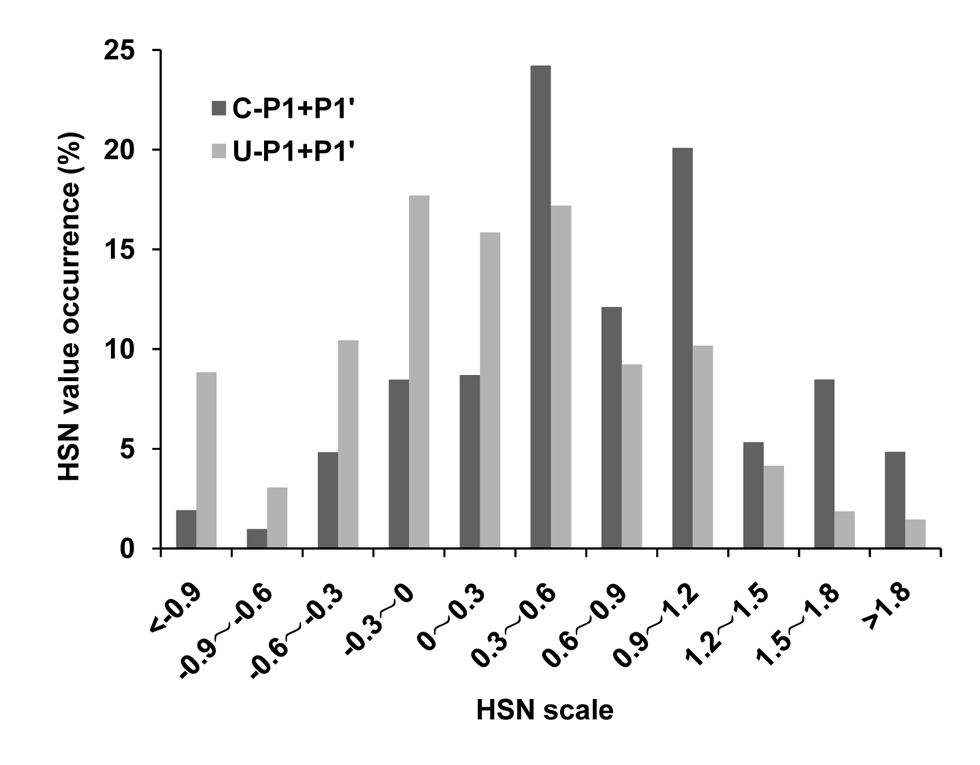

Supplement: Figure S1 — Comparison of the HSN distributions between the cleaved and undetected peptides in the CCPD. The hydrophobic values of amino acids were analyzed according to Wimley method [38]. (TIF) [file pone.0065733.s001.tif]
