# Supplementary material for: Proteolytic Characteristics of Cathepsin D Related to the Recognition and Cleavage of Its Target Proteins
Source: PLoS One. 2013 Jun 20;8(6):e65733. doi: 10.1371/journal.pone.0065733 (PMC3688724; doi:10.1371/journal.pone.0065733)
Supplement: Table S1 — The tryptic BSA peptides identified via LC-MS/MS and no enzyme search in Mascot. (DOC) [file pone.0065733.s002.doc]

**Table S1. The tryptic BSA peptides identified via LC-MS/MS and no enzyme search in Mascot.**

Columns from left to right contain: number, start and end position of the identified peptide in BSA, the peptide sequence identified by LC-MS/MS.

| **Number** | **Start** | **End** | **Peptide sequence** |
| --- | --- | --- | --- |
| 1 | 66 | 75 | LVNELTEFAK |
| 2 | 76 | 88 | TCVADESHAGCEK |
| 3 | 89 | 100 | SLHTLFGDELCK |
| 4 | 106 | 117 | ETYGDMADCCEK |
| 5 | 139 | 151 | LKPDPNTLCDEFK |
| 6 | 161 | 167 | YLYEIAR |
| 7 | 184 | 197 | YNGVFQECCQAEDK |
| 8 | 249 | 256 | AEFVEVTK |
| 9 | 267 | 280 | ECCHGDLLECADDR |
| 10 | 286 | 297 | YICDNQDTISSK |
| 11 | 319 | 340 | DAIPENLPPLTADFAEDKDVCK |
| 12 | 347 | 359 | DAFLGSFLYEYSR |
| 13 | 375 | 386 | EYEATLEECCAK |
| 14 | 387 | 399 | DDPHACYSTVFDK |
| 15 | 402 | 412 | HLVDEPQNLIK |
| 16 | 413 | 420 | QNCDQFEK |
| 17 | 421 | 433 | LGEYGFQNALIVR |
| 18 | 438 | 451 | VPQVSTPTLVEVSR |
| 19 | 469 | 482 | MPCTEDYLSLILNR |
| 20 | 499 | 507 | CCTESLVNR |
| 21 | 549 | 557 | QTALVELLK |
| 22 | 569 | 580 | TVMENFVAFVDK |
| 23 | 581 | 597 | CCAADDKEACFAVEGPK |
| 24 | 588 | 597 | EACFAVEGPK |
