# Supplementary material for: Proteolytic Characteristics of Cathepsin D Related to the Recognition and Cleavage of Its Target Proteins
Source: PLoS One. 2013 Jun 20;8(6):e65733. doi: 10.1371/journal.pone.0065733 (PMC3688724; doi:10.1371/journal.pone.0065733)
Supplement: Table S2 — CD-cleaved peptide database (CCPD). (DOC) [file pone.0065733.s003.doc]

**Table S2. CD-cleaved peptide database (CCPD).**

Columns from left to right contain: protein name, start and end position of the identified peptide in each protein, the peptide sequence identified by LC-MS/MS.

| **Protein** | **Start** | **End** | **Peptide sequence** |
| --- | --- | --- | --- |
| BSA | 25 | 37 | DTHKSEIAHRFKD |
|  | 25 | 38 | DTHKSEIAHRFKDL |
|  | 25 | 45 | DTHKSEIAHRFKDLGEEHFKG |
|  | 25 | 48 | DTHKSEIAHRFKDLGEEHFKGLVL |
|  | 48 | 53 | LIAFSQ |
|  | 49 | 55 | IAFSQYL |
|  | 55 | 63 | LQQCPFDEH |
|  | 55 | 70 | LQQCPFDEHVKLVNEL |
|  | 62 | 70 | EHVKLVNEL |
|  | 63 | 70 | HVKLVNEL |
|  | 63 | 72 | HVKLVNELTE |
|  | 64 | 72 | VKLVNELTE |
|  | 131 | 136 | DDSPDL |
|  | 151 | 161 | KADEKKFWGKY |
|  | 151 | 162 | KADEKKFWGKYL |
|  | 163 | 174 | YEIARRHPYFYA |
|  | 165 | 174 | IARRHPYFYA |
|  | 165 | 177 | IARRHPYFYAPEL |
|  | 165 | 178 | IARRHPYFYAPELL |
|  | 179 | 188 | YYANKYNGVF |
|  | 180 | 188 | YANKYNGVF |
|  | 237 | 246 | WSVARLSQKF |
|  | 237 | 250 | WSVARLSQKFPKAE |
|  | 237 | 251 | WSVARLSQKFPKAEF |
|  | 237 | 253 | WSVARLSQKFPKAEFVE |
|  | 241 | 250 | RLSQKFPKAE |
|  | 241 | 251 | RLSQKFPKAEF |
|  | 243 | 250 | SQKFPKAE |
|  | 243 | 251 | SQKFPKAEF |
|  | 251 | 257 | FVEVTKL |
|  | 251 | 261 | FVEVTKLVTDL |
|  | 252 | 257 | VEVTKL |
|  | 252 | 260 | VEVTKLVTD |
|  | 252 | 261 | VEVTKLVTDL |
|  | 320 | 331 | AIPENLPPLTAD |
|  | 348 | 354 | FLGSFL |
|  | 355 | 369 | YEYSRRHPEYAVSVL |
|  | 357 | 369 | YSRRHPEYAVSVL |
|  | 370 | 380 | LRLAKEYEATL |
|  | 398 | 410 | DKLKHLVDEPQNL |
|  | 419 | 430 | EKLGEYGFQNAL |
|  | 422 | 429 | GEYGFQNA |
|  | 422 | 430 | GEYGFQNAL |
|  | 429 | 446 | LIVRYTRKVPQVSTPTL |
|  | 431 | 446 | IVRYTRKVPQVSTPTL |
|  | 434 | 446 | YTRKVPQVSTPTL |
|  | 435 | 446 | TRKVPQVSTPTL |
|  | 519 | 525 | TYVPKAF |
|  | 519 | 529 | TYVPKAFDEKL |
|  | 520 | 529 | YVPKAFDEKL |
|  | 553 | 567 | VELLKHKPKATEEQL |
|  | 553 | 569 | VELLKHKPKATEEQLKT |
|  | 553 | 573 | VELLKHKPKATEEQLKTVMEN |
|  | 553 | 574 | VELLKHKPKATEEQLKTVMENF |
|  | 556 | 569 | LKHKPKATEEQLKT |
|  | 556 | 574 | LKHKPKATEEQLKTVMENF |
|  | 557 | 573 | KHKPKATEEQLKTVMEN |
|  | 568 | 574 | KTVMENF |
|  | 592 | 598 | AVEGPKL |
|  | 596 | 607 | PKLVVSTQTALA |
|  | 598 | 603 | LVVSTQ |
|  | 598 | 605 | LVVSTQTA |
|  | 598 | 607 | LVVSTQTALA |
|  | 599 | 607 | VVSTQTALA |
| HSA | 25 | 38 | DAHKSEVAHRFKDL |
|  | 25 | 42 | DAHKSEVAHRFKDLGEEN |
|  | 25 | 43 | DAHKSEVAHRFKDLGEENF |
|  | 25 | 45 | DAHKSEVAHRFKDLGEENFKA |
|  | 25 | 46 | DAHKSEVAHRFKDLGEENFKAL |
|  | 26 | 45 | AHKSEVAHRFKDLGEENFKA |
|  | 27 | 43 | HKSEVAHRFKDLGEENF |
|  | 27 | 45 | HKSEVAHRFKDLGEENFKA |
|  | 59 | 72 | PFEDHVKLVNEVTE |
|  | 61 | 72 | EDHVKLVNEVTE |
|  | 64 | 72 | VKLVNEVTE |
|  | 64 | 73 | VKLVNEVTEF |
|  | 67 | 72 | VNEVTE |
|  | 152 | 158 | HDNEETF |
|  | 235 | 252 | FKAWAVARLSQRFPKAEF |
|  | 236 | 252 | KAWAVARLSQRFPKAEF |
|  | 239 | 247 | AVARLSQRF |
|  | 244 | 252 | SQRFPKAEF |
|  | 244 | 262 | SQRFPKAEFAEVSKLVTDL |
|  | 248 | 262 | PKAEFAEVSKLVTDL |
|  | 252 | 262 | FAEVSKLVTDL |
|  | 253 | 261 | AEVSKLVTD |
|  | 253 | 262 | AEVSKLVTDL |
|  | 355 | 365 | LYEYARRHPDY |
|  | 355 | 369 | LYEYARRHPDYSVVL |
|  | 358 | 369 | YARRHPDYSVVL |
|  | 399 | 407 | DEFKPLVEE |
|  | 399 | 411 | DEFKPLVEEPQNL |
|  | 401 | 411 | FKPLVEEPQNL |
|  | 402 | 411 | KPLVEEPQNL |
|  | 419 | 431 | FEQLGEYKFQNAL |
|  | 426 | 434 | KFQNALLVR |
|  | 431 | 447 | LLVRYTKKVPQVSTPTL |
|  | 432 | 447 | LVRYTKKVPQVSTPTL |
|  | 432 | 449 | LVRYTKKVPQVSTPTLVE |
|  | 433 | 447 | VRYTKKVPQVSTPTL |
|  | 435 | 447 | YTKKVPQVSTPTL |
|  | 436 | 447 | TKKVPQVSTPTL |
|  | 516 | 526 | EVDETYVPKEF |
|  | 518 | 526 | DETYVPKEF |
|  | 519 | 526 | ETYVPKEF |
|  | 520 | 526 | TYVPKEF |
|  | 520 | 530 | TYVPKEFNAET |
|  | 520 | 531 | TYVPKEFNAETF |
|  | 520 | 532 | TYVPKEFNAETFT |
|  | 520 | 533 | TYVPKEFNAETFTF |
|  | 527 | 531 | NAETF |
|  | 527 | 533 | NAETFTF |
|  | 557 | 574 | VKHKPKATKEQLKAVMDD |
|  | 557 | 575 | VKHKPKATKEQLKAVMDDF |
|  | 600 | 609 | VAASQAALGL |
| PSA | 25 | 38 | DTYKSEIAHRFKDL |
|  | 25 | 41 | DTYKSEIAHRFKDLGEQ |
|  | 25 | 42 | DTYKSEIAHRFKDLGEQY |
|  | 25 | 45 | DTYKSEIAHRFKDLGEQYFKG |
|  | 25 | 48 | DTYKSEIAHRFKDLGEQYFKGLVL |
|  | 29 | 41 | SEIAHRFKDLGEQ |
|  | 29 | 45 | SEIAHRFKDLGEQYFKG |
|  | 39 | 45 | GEQYFKG |
|  | 42 | 48 | YFKGLVL |
|  | 43 | 48 | FKGLVL |
|  | 46 | 50 | LVLIA |
|  | 64 | 69 | VFLGTF |
|  | 64 | 72 | VKLVREVTE |
|  | 64 | 73 | VKLVREVTEF |
|  | 66 | 72 | LVREVTE |
|  | 67 | 73 | VREVTEF |
|  | 163 | 174 | YEIARRHPYFYA |
|  | 163 | 177 | YEIARRHPYFYAPEL |
|  | 178 | 188 | LYYAIIYKDVF |
|  | 179 | 187 | YYAIIYKDV |
|  | 179 | 188 | YYAIIYKDVF |
|  | 180 | 188 | YAIIYKDVF |
|  | 181 | 188 | AIIYKDVF |
|  | 240 | 251 | ARLSQRFPKADF |
|  | 240 | 261 | ARLSQRFPKADFTEISKIVTDL |
|  | 243 | 251 | SQRFPKADF |
|  | 243 | 253 | SQRFPKADFTE |
|  | 243 | 261 | SQRFPKADFTEISKIVTDL |
|  | 244 | 261 | QRFPKADFTEISKIVTDL |
|  | 246 | 261 | FPKADFTEISKIVTDL |
|  | 249 | 256 | ADFTEISK |
|  | 250 | 261 | DFTEISKIVTDL |
|  | 251 | 261 | FTEISKIVTDL |
|  | 252 | 261 | TEISKIVTDL |
|  | 257 | 263 | IVTDLAK |
|  | 322 | 331 | PADLNPLEHD |
|  | 355 | 368 | YEYSRRHPDYSVSL |
|  | 369 | 379 | LLRIAKIYEAT |
|  | 370 | 377 | LRIAKIYE |
|  | 398 | 408 | DKFQPLVDEPK |
|  | 398 | 409 | DKFQPLVDEPKN |
|  | 398 | 410 | DKFQPLVDEPKNL |
|  | 399 | 410 | KFQPLVDEPKNL |
|  | 422 | 429 | GEYGFQNA |
|  | 422 | 430 | GEYGFQNAL |
|  | 428 | 436 | NALIVRYTK |
|  | 429 | 436 | ALIVRYTK |
|  | 430 | 446 | LIVRYTKKVPQVSTPTL |
|  | 430 | 448 | LIVRYTKKVPQVSTPTLVE |
|  | 431 | 446 | IVRYTKKVPQVSTPTL |
|  | 434 | 446 | YTKKVPQVSTPTL |
|  | 437 | 446 | KVPQVSTPTL |
|  | 514 | 524 | LTPDETYKPKE |
|  | 515 | 525 | TPDETYKPKEF |
|  | 519 | 525 | TYKPKEF |
|  | 526 | 532 | VEGTFTF |
|  | 553 | 567 | VELLKHKPHATEEQL |
|  | 553 | 568 | VELLKHKPHATEEQLR |
|  | 553 | 571 | VELLKHKPHATEEQLRTVL |
|  | 555 | 567 | LLKHKPHATEEQL |
|  | 556 | 567 | LKHKPHATEEQL |
|  | 556 | 571 | LKHKPHATEEQLRTVL |
|  | 556 | 574 | LKHKPHATEEQLRTVLGNF |
|  | 557 | 567 | KHKPHATEEQL |
|  | 568 | 574 | RTVLGNF |
|  | 568 | 576 | RTVLGNFAA |
|  | 569 | 574 | TVLGNF |
|  | 592 | 598 | AVEGPKF |
|  | 592 | 599 | AVEGPKFV |
|  | 598 | 607 | FVIEIRGILA |
|  | 599 | 606 | VIEIRGIL |
|  | 599 | 607 | VIEIRGILA |
|  | 600 | 607 | IEIRGILA |
| D-OVA | 20 | 28 | KVHHANENI |
|  | 32 | 38 | PIAIMSA |
|  | 44 | 60 | LGAKDSTRTQINKVVRF |
|  | 45 | 55 | GAKDSTRTQIN |
|  | 45 | 60 | GAKDSTRTQINKVVRF |
|  | 61 | 68 | DKLPGFGD |
|  | 61 | 71 | DKLPGFGDSIE |
|  | 87 | 99 | ILNQITKPNDVYS |
|  | 89 | 99 | NQITKPNDVYS |
|  | 89 | 100 | NQITKPNDVYSF |
|  | 89 | 101 | NQITKPNDVYSFS |
|  | 89 | 102 | NQITKPNDVYSFSL |
|  | 91 | 98 | ITKPNDVY |
|  | 91 | 99 | ITKPNDVYS |
|  | 91 | 100 | ITKPNDVYSF |
|  | 91 | 101 | ITKPNDVYSFS |
|  | 91 | 102 | ITKPNDVYSFSL |
|  | 101 | 106 | SLASRL |
|  | 103 | 118 | ASRLYAEERYPILPEY |
|  | 107 | 112 | YAEERY |
|  | 107 | 115 | YAEERYPIL |
|  | 107 | 118 | YAEERYPILPEY |
|  | 108 | 118 | AEERYPILPEY |
|  | 116 | 120 | PEYLQ |
|  | 122 | 135 | VKELYRGGLEPINF |
|  | 126 | 135 | YRGGLEPINF |
|  | 135 | 145 | FQTAADQAREL |
|  | 135 | 148 | FQTAADQARELINS |
|  | 135 | 149 | FQTAADQARELINSW |
|  | 136 | 145 | QTAADQAREL |
|  | 136 | 148 | QTAADQARELINS |
|  | 136 | 149 | QTAADQARELINSW |
|  | 139 | 148 | ADQARELINS |
|  | 139 | 149 | ADQARELINSW |
|  | 149 | 160 | WVESQTNGIIRN |
|  | 149 | 172 | WVESQTNGIIRNVLQPSSVDSQTA |
|  | 150 | 160 | VESQTNGIIRN |
|  | 150 | 172 | VESQTNGIIRNVLQPSSVDSQTA |
|  | 152 | 160 | SQTNGIIRN |
|  | 157 | 168 | IIRNVLQPSSVD |
|  | 157 | 172 | IIRNVLQPSSVDSQTA |
|  | 160 | 172 | NVLQPSSVDSQTA |
|  | 161 | 172 | VLQPSSVDSQTA |
|  | 161 | 173 | VLQPSSVDSQTAM |
|  | 161 | 175 | VLQPSSVDSQTAMVL |
|  | 173 | 178 | MVLVNA |
|  | 176 | 181 | VNAIVF |
|  | 176 | 185 | VNAIVFKGLW |
|  | 179 | 185 | IVFKGLW |
|  | 189 | 197 | FKDEDTQAM |
|  | 189 | 199 | FKDEDTQAMPF |
|  | 195 | 204 | QAMPFRVTEQ |
|  | 205 | 214 | ESKPVQMMYQ |
|  | 219 | 233 | RVASMASEKMKILEL |
|  | 234 | 240 | PFASGTM |
|  | 243 | 256 | LVLLPDEVSGLEQL |
|  | 244 | 255 | VLLPDEVSGLEQ |
|  | 244 | 256 | VLLPDEVSGLEQL |
|  | 246 | 256 | LPDEVSGLEQL |
|  | 247 | 253 | PDEVSGL |
|  | 247 | 256 | PDEVSGLEQL |
|  | 263 | 268 | EKLTEW |
|  | 263 | 271 | EKLTEWTSS |
|  | 263 | 272 | EKLTEWTSSN |
|  | 263 | 273 | EKLTEWTSSNV |
|  | 263 | 275 | EKLTEWTSSNVME |
|  | 263 | 276 | EKLTEWTSSNVMEE |
|  | 266 | 275 | TEWTSSNVME |
|  | 301 | 306 | MGITDV |
|  | 308 | 318 | SSSANLSGISS |
|  | 308 | 322 | SSSANLSGISSAESL |
|  | 308 | 323 | SSSANLSGISSAESLK |
|  | 308 | 325 | SSSANLSGISSAESLKIS |
|  | 308 | 326 | SSSANLSGISSAESLKISQ |
|  | 308 | 337 | SSSANLSGISSAESLKISQAVHAAHAEINE |
|  | 314 | 326 | SGISSAESLKISQ |
|  | 316 | 326 | ISSAESLKISQ |
|  | 319 | 337 | AESLKISQAVHAAHAEINE |
|  | 323 | 337 | KISQAVHAAHAEINE |
|  | 327 | 337 | AVHAAHAEINE |
|  | 338 | 351 | AGREVVGSAEAGVD |
|  | 338 | 352 | AGREVVGSAEAGVDA |
|  | 338 | 358 | AGREVVGSAEAGVDAASVSEE |
|  | 359 | 367 | FRADHPFLF |
|  | 360 | 366 | RADHPFL |
|  | 360 | 367 | RADHPFLF |
|  | 369 | 379 | IKHIATNAVLF |
| TF | 74 | 81 | NEADAVTL |
|  | 77 | 81 | DAVTL |
|  | 78 | 88 | AVTLDAGLVYD |
|  | 78 | 89 | AVTLDAGLVYDA |
|  | 82 | 87 | DAGLVY |
|  | 82 | 88 | DAGLVYD |
|  | 82 | 89 | DAGLVYDA |
|  | 82 | 90 | DAGLVYDAY |
|  | 83 | 89 | AGLVYDA |
|  | 85 | 89 | LVYDA |
|  | 88 | 102 | DAYLAPNNLKPVVAE |
|  | 88 | 103 | DAYLAPNNLKPVVAEF |
|  | 89 | 101 | AYLAPNNLKPVVA |
|  | 89 | 102 | AYLAPNNLKPVVAE |
|  | 89 | 103 | AYLAPNNLKPVVAEF |
|  | 90 | 101 | YLAPNNLKPVVA |
|  | 90 | 102 | YLAPNNLKPVVAE |
|  | 90 | 103 | YLAPNNLKPVVAEF |
|  | 90 | 114 | YLAPNNLKPVVAEFYGSKEDPQTFY |
|  | 91 | 102 | LAPNNLKPVVAE |
|  | 91 | 103 | LAPNNLKPVVAEF |
|  | 103 | 113 | FYGSKEDPQTF |
|  | 103 | 114 | FYGSKEDPQTFY |
|  | 104 | 113 | YGSKEDPQTF |
|  | 104 | 114 | YGSKEDPQTFY |
|  | 104 | 115 | YGSKEDPQTFYY |
|  | 104 | 116 | YGSKEDPQTFYYA |
|  | 105 | 114 | GSKEDPQTFY |
|  | 110 | 114 | PQTFY |
|  | 115 | 128 | YAVAVVKKDSGFQM |
|  | 221 | 230 | VAFVKHSTIF |
|  | 223 | 230 | FVKHSTIF |
|  | 223 | 232 | FVKHSTIFEN |
|  | 223 | 233 | FVKHSTIFENL |
|  | 224 | 233 | VKHSTIFENL |
|  | 231 | 245 | ENLANKADRDQYELL |
|  | 234 | 244 | ANKADRDQYEL |
|  | 284 | 303 | ELLNQAQEHFGKDKSKEFQL |
|  | 285 | 303 | LLNQAQEHFGKDKSKEFQL |
|  | 286 | 303 | LNQAQEHFGKDKSKEFQL |
|  | 287 | 303 | NQAQEHFGKDKSKEFQL |
|  | 289 | 303 | AQEHFGKDKSKEFQL |
|  | 304 | 313 | FSSPHGKDLL |
|  | 314 | 321 | FKDSAHGF |
|  | 314 | 337 | FKDSAHGFLKVPPRMDAKMYLGYE |
|  | 336 | 341 | YEYVTA |
|  | 406 | 414 | DAMSLDGGF |
|  | 409 | 414 | SLDGGF |
|  | 551 | 573 | FVKHQTVPQNTGGKNPDPWAKNL |
|  | 554 | 571 | HQTVPQNTGGKNPDPWAK |
|  | 641 | 648 | FRSETKDL |
|  | 641 | 649 | FRSETKDLL |
|  | 658 | 673 | AKLHDRNTYEKYLGEE |
|  | 674 | 681 | YVKAVGNL |
| HB | 1 | 13 | VLSAADKANVKAA |
|  | 1 | 15 | VLSAADKANVKAAWG |
|  | 1 | 21 | VLSAADKANVKAAWGKVGGQA |
|  | 1 | 22 | VLSAADKANVKAAWGKVGGQAG |
|  | 2 | 15 | VHLSAEEKEAVLGL |
|  | 2 | 32 | VHLSAEEKEAVLGLWGKVNVDEVGGEALGRL |
|  | 15 | 32 | GKVGGQAGAHGAEALERM |
|  | 16 | 32 | WGKVNVDEVGGEALGRL |
|  | 16 | 32 | GKVNVDEVGGEALGRL |
|  | 16 | 33 | WGKVNVDEVGGEALGRLL |
|  | 21 | 32 | VDEVGGEALGRL |
|  | 23 | 32 | EVGGEALGRL |
|  | 23 | 33 | EVGGEALGRLL |
|  | 24 | 32 | VGGEALGRL |
|  | 32 | 42 | LLVVYPWTQRF |
|  | 33 | 42 | LVVYPWTQRF |
|  | 33 | 46 | FLGFPTTKTYFPHF |
|  | 33 | 51 | FLGFPTTKTYFPHFNLSHG |
|  | 33 | 53 | FLGFPTTKTYFPHFNLSHGSD |
|  | 34 | 46 | LGFPTTKTYFPHF |
|  | 87 | 98 | HAHKLRVDPVNF |
|  | 95 | 105 | PVNFKLLSHCL |
|  | 105 | 112 | RLLGNVIV |
|  | 106 | 115 | LVTLAAHHPD |
|  | 106 | 116 | LVTLAAHHPDD |
|  | 106 | 128 | LVTLAAHHPDDFNPSVHASLDKF |
|  | 106 | 131 | LVTLAAHHPDDFNPSVHASLDKFLAN |
|  | 107 | 122 | VTLAAHHPDDFNPSVH |
|  | 109 | 115 | NVIVVVL |
|  | 109 | 122 | LAAHHPDDFNPSVH |
|  | 109 | 136 | LAAHHPDDFNPSVHASLDKFLANVSTVL |
|  | 110 | 122 | AAHHPDDFNPSVH |
|  | 110 | 123 | AAHHPDDFNPSVHA |
|  | 110 | 125 | AAHHPDDFNPSVHASL |
|  | 110 | 126 | AAHHPDDFNPSVHASLD |
|  | 110 | 128 | AAHHPDDFNPSVHASLDKF |
|  | 114 | 128 | PDDFNPSVHASLDKF |
|  | 116 | 128 | DFNPSVHASLDKF |
|  | 117 | 128 | FNPSVHASLDKF |
|  | 129 | 141 | LANVSTVLTSKYR |
|  | 130 | 141 | ANVSTVLTSKYR |
|  | 131 | 138 | FQKVVAGV |
|  | 131 | 147 | FQKVVAGVANALAHKYH |
| AKR1A1 | 8 | 19 | LHTGQKMPLIGL |
|  | 8 | 21 | LHTGQKMPLIGLGT |
|  | 23 | 40 | KSEPGQVKAAVKYALSVG |
|  | 47 | 60 | AAIYGNEPEIGEAL |
|  | 59 | 75 | ALKEDVGPGKAVPREEL |
|  | 60 | 75 | LKEDVGPGKAVPREEL |
|  | 61 | 75 | KEDVGPGKAVPREEL |
|  | 61 | 76 | KEDVGPGKAVPREELF |
|  | 82 | 99 | WNTKHHPEDVEPALRKTL |
|  | 174 | 183 | SVASVRPAVL |
|  | 184 | 196 | QVECHPYLAQNEL |
|  | 209 | 228 | AYSPLGSSDRAWRDPDEPVL |
|  | 236 | 249 | ALAEKYGRSPAQIL |
|  | 237 | 249 | LAEKYGRSPAQIL |
|  | 238 | 249 | AEKYGRSPAQIL |
|  | 278 | 289 | DFTFSPEEMKQL |
|  | 279 | 289 | FTFSPEEMKQL |
|  | 290 | 296 | NALNKNW |
|  | 290 | 298 | NALNKNWRY |
| AKR1B10 | 4 | 18 | FVELSTKAKMPIVGL |
|  | 4 | 20 | VELSTKAKMPIVGLGT |
|  | 4 | 20 | FVELSTKAKMPIVGLGT |
|  | 7 | 18 | LSTKAKMPIVGL |
|  | 7 | 20 | LSTKAKMPIVGLGT |
|  | 8 | 18 | STKAKMPIVGL |
|  | 36 | 44 | IDAGYRHID |
|  | 36 | 46 | IDAGYRHIDCA |
|  | 36 | 47 | IDAGYRHIDCAY |
|  | 40 | 47 | YRHIDCAY |
|  | 47 | 62 | YVYQNEHEVGEAIQEK |
|  | 48 | 58 | VYQNEHEVGEA |
|  | 48 | 61 | VYQNEHEVGEAIQE |
|  | 48 | 62 | VYQNEHEVGEAIQEK |
|  | 48 | 66 | VYQNEHEVGEAIQEKIQEK |
|  | 53 | 62 | HEVGEAIQEK |
|  | 59 | 73 | IQEKIQEKAVKREDL |
|  | 63 | 74 | IQEKAVKREDLF |
|  | 74 | 83 | FIVSKLWPTF |
|  | 75 | 83 | IVSKLWPTF |
|  | 78 | 83 | KLWPTF |
|  | 84 | 99 | FERPLVRKAFEKTLKD |
|  | 84 | 102 | FERPLVRKAFEKTLKDLKL |
|  | 110 | 123 | IHWPQGFKSGDDLF |
|  | 124 | 139 | PKDDKGNAIGGKATFL |
|  | 124 | 141 | PKDDKGNAIGGKATFLDA |
|  | 131 | 135 | AIGGK |
|  | 146 | 157 | EELVDEGLVKAL |
|  | 148 | 157 | LVDEGLVKAL |
|  | 148 | 162 | LVDEGLVKALGVSNF |
|  | 149 | 156 | VDEGLVKA |
|  | 149 | 157 | VDEGLVKAL |
|  | 149 | 161 | VDEGLVKALGVSN |
|  | 149 | 162 | VDEGLVKALGVSNF |
|  | 158 | 170 | GVSNFSHFQIEKL |
|  | 171 | 183 | LNKPGLKYKPVTN |
|  | 171 | 184 | LNKPGLKYKPVTNQ |
|  | 197 | 208 | IQYCHSKGITVT |
|  | 197 | 209 | IQYCHSKGITVTA |
|  | 210 | 228 | YSPLGSPDRPWAKPEDPSL |
|  | 232 | 249 | PKIKEIAAKHKKTAAQVL |
|  | 237 | 249 | IAAKHKKTAAQVL |
|  | 250 | 273 | IRFHIQRNVIVIPKSVTPARIVEN |
|  | 253 | 274 | HIQRNVIVIPKSVTPARIVENI |
|  | 258 | 273 | VIVIPKSVTPARIVEN |
|  | 258 | 274 | VIVIPKSVTPARIVENI |
|  | 273 | 279 | NIQVFDF |
|  | 279 | 286 | FKLSDEEM |
|  | 279 | 288 | FKLSDEEMAT |
|  | 280 | 290 | KLSDEEMATIL |
|  | 280 | 292 | KLSDEEMATILSF |
|  | 288 | 295 | TILSFNRN |
|  | 288 | 296 | TILSFNRNW |
| AKR1C1 | 21 | 40 | FGTYAPAEVPKSKALEATKL |
|  | 22 | 40 | GTYAPAEVPKSKALEATKL |
|  | 25 | 40 | APAEVPKSKALEATKL |
|  | 26 | 40 | PAEVPKSKALEATKL |
|  | 46 | 58 | FRHIDSAHLYNNE |
|  | 46 | 62 | FRHIDSAHLYNNEEQVG |
|  | 63 | 80 | LAIRSKIADGSVKREDIF |
|  | 269 | 285 | AKSYNEQRIRQNVQVFE |
|  | 287 | 302 | QLTSEEMKAIDGLNRN |
|  | 289 | 305 | TSEEMKAIDGLNRNVRY |
| AKR1C3 | 21 | 35 | FGTYAPPEVPRSKAL |
|  | 21 | 40 | FGTYAPPEVPRSKALEVTKL |
|  | 25 | 40 | APPEVPRSKALEVTKL |
|  | 46 | 62 | FRHIDSAHLYNNEEQVG |
|  | 63 | 80 | LAIRSKIADGSVKREDIF |
|  | 116 | 140 | IHSPMSLKPGEELSPTDENGKVIFD |
|  | 176 | 189 | ILNKPGLKYKPVCN |
|  | 205 | 213 | FCKSKDIVL |
|  | 214 | 235 | VAYSALGSQRDKRWVDPNSPVL |
|  | 258 | 268 | RYQLQRGVVVL |
|  | 269 | 285 | AKSYNEQRIRQNVQVFE |
|  | 269 | 286 | AKSYNEQRIRQNVQVFEF |
|  | 286 | 299 | FQLTAEDMKAIDGL |
|  | 287 | 299 | QLTAEDMKAIDGL |
|  | 300 | 306 | DRNLHYF |
|  | 307 | 323 | NSDSFASHPNYPYSDEY |
|  | 312 | 322 | ASHPNYPYSDE |
|  | 312 | 323 | ASHPNYPYSDEY |
| GST-A3 | 24 | 33 | AAAGVEFEEK |
|  | 24 | 34 | AAAGVEFEEKF |
| GST-M1 | 61 | 74 | PYLIDGSHKITQSN |
|  | 99 | 111 | IVENQVMDTRMQL |
|  | 138 | 150 | YSEFLGKRPWFAG |
| GST-P1 | 20 | 32 | LLADQGQSWKEE |
|  | 22 | 32 | LADQGQSWKEE |
|  | 92 | 104 | MVNDGVEDLRGKY |
|  | 101 | 107 | RGKYVTL |
|  | 134 | 143 | LSQNQGGKAF |
|  | 134 | 148 | LSQNQGGKAFIVGDQ |
|  | 134 | 151 | LSQNQGGKAFIVGDQISF |
|  | 144 | 151 | IVGDQISF |
|  | 175 | 180 | PLLSAY |
| CD | 65 | 73 | GPIPEVLKN |
|  | 65 | 74 | GPIPEVLKNY |
|  | 75 | 79 | MDAQY |
|  | 139 | 149 | DIHYGSGSLSG |
|  | 139 | 150 | DIHYGSGSLSGY |
|  | 177 | 190 | QVFGEATKQPGITF |
|  | 180 | 190 | GEATKQPGITF |
|  | 201 | 209 | MAYPRISVN |
|  | 201 | 212 | MAYPRISVNNVL |
|  | 204 | 212 | PRISVNNVL |
|  | 219 | 227 | MQQKLVDQN |
|  | 219 | 229 | MQQKLVDQNIF |
|  | 219 | 230 | MQQKLVDQNIFS |
|  | 219 | 231 | MQQKLVDQNIFSF |
|  | 232 | 245 | YLSRDPDAQPGGEL |
|  | 232 | 246 | YLSRDPDAQPGGELM |
|  | 232 | 254 | YLSRDPDAQPGGELMLGGTDSKY |
|  | 233 | 245 | LSRDPDAQPGGEL |
|  | 233 | 247 | LSRDPDAQPGGELML |
|  | 233 | 253 | LSRDPDAQPGGELMLGGTDSK |
|  | 234 | 245 | SRDPDAQPGGEL |
|  | 246 | 254 | MLGGTDSKY |
|  | 301 | 310 | MVGPVDEVRE |
|  | 301 | 311 | MVGPVDEVREL |
|  | 301 | 313 | MVGPVDEVRELQK |
|  | 308 | 324 | VRELQKAIGAVPLIQGE |
|  | 312 | 324 | QKAIGAVPLIQGE |
|  | 315 | 324 | IGAVPLIQGE |
|  | 341 | 353 | KLGGKGYKLSPED |
|  | 341 | 356 | KLGGKGYKLSPEDYTL |
|  | 394 | 410 | YTVFDRDNNRVGFAEAA |
|  | 398 | 406 | DRDNNRVGF |
|  | 398 | 410 | DRDNNRVGFAEAA |
|  | 398 | 411 | DRDNNRVGFAEAAR |
