# Supplementary material for: Proteolytic Characteristics of Cathepsin D Related to the Recognition and Cleavage of Its Target Proteins
Source: PLoS One. 2013 Jun 20;8(6):e65733. doi: 10.1371/journal.pone.0065733 (PMC3688724; doi:10.1371/journal.pone.0065733)
Supplement: Table S3 — The corresponding occurrence frequencies of the residues at each position in Figure 1B. (DOC) [file pone.0065733.s004.doc]

**Table S3. The corresponding occurrence frequencies of the residues at each position in Figure 1B.**

| **P6** | **%** | **P5** | **%** | **P4** | **%** | **P3** | **%** | **P2** | **%** | **P1** | **%** | **P1'** | **%** | **P2'** | **%** | **P3'** | **%** | **P4'** | **%** | **P5'** | **%** | **P6'** | **%** |
| --- | --- | --- | --- | --- | --- | --- | --- | --- | --- | --- | --- | --- | --- | --- | --- | --- | --- | --- | --- | --- | --- | --- | --- |
| A | 15 | L | 12 | V | 12 | E | 11 | K | 17 | L | 36 | L | 17 | E | 18 | L | 14 | K | 11 | K | 17 | K | 17 |
| K | 14 | F | 12 | E | 11 | L | 11 | E | 14 | F | 13 | V | 14 | K | 14 | V | 14 | L | 9 | F | 11 | D | 9 |
| F | 8 | A | 11 | F | 9 | F | 9 | V | 11 | E | 10 | Y | 12 | V | 14 | K | 12 | V | 9 | P | 9 | L | 9 |
| D | 6 | E | 11 | P | 9 | P | 9 | D | 9 | A | 9 | F | 9 | A | 11 | E | 11 | A | 8 | A | 8 | F | 8 |
| C | 6 | K | 8 | A | 8 | K | 8 | A | 8 | D | 7 | I | 6 | T | 9 | A | 6 | S | 8 | H | 8 | T | 8 |
| E | 6 | P | 8 | L | 8 | S | 8 | Y | 8 | Y | 6 | P | 6 | Y | 6 | R | 6 | E | 6 | V | 8 | R | 6 |
| Q | 6 | Y | 6 | G | 6 | T | 8 | L | 6 | G | 3 | T | 6 | Q | 5 | F | 6 | P | 6 | R | 6 | Q | 6 |
| T | 6 | S | 5 | S | 6 | A | 6 | T | 6 | K | 3 | D | 5 | I | 5 | C | 5 | R | 5 | T | 6 | P | 6 |
| Y | 6 | T | 5 | C | 5 | D | 6 | N | 5 | T | 3 | E | 5 | L | 5 | Q | 5 | N | 5 | N | 5 | Y | 6 |
| V | 6 | V | 5 | Q | 5 | Q | 6 | Q | 5 | R | 2 | K | 5 | R | 3 | S | 5 | C | 5 | C | 5 | V | 6 |
| G | 5 | R | 3 | K | 3 | G | 6 | R | 3 | N | 2 | A | 3 | H | 3 | Y | 5 | G | 5 | Q | 5 | A | 5 |
| H | 5 | N | 3 | T | 3 | V | 6 | F | 3 | Q | 2 | Q | 3 | S | 3 | D | 3 | H | 5 | Y | 5 | N | 5 |
| L | 3 | Q | 3 | W | 3 | N | 3 | C | 2 | H | 2 | G | 3 | D | 2 | P | 3 | F | 5 | D | 3 | E | 5 |
| R | 2 | D | 2 | Y | 3 | I | 2 | H | 2 | V | 2 | R | 2 | G | 2 | N | 2 | T | 5 | E | 3 | S | 3 |
| N | 2 | C | 2 | R | 2 | M | 2 | P | 2 |  |  | H | 2 | M | 2 | G | 2 | Q | 3 | L | 2 | H | 2 |
| I | 2 | G | 2 | N | 2 |  |  | S | 2 |  |  | S | 2 |  |  | H | 2 | I | 3 | S | 2 |  |  |
| P | 2 | H | 2 | D | 2 |  |  |  |  |  |  | W | 2 |  |  | I | 2 | D | 2 |  |  |  |  |
| W | 2 | I | 2 | H | 2 |  |  |  |  |  |  |  |  |  |  |  |  | M | 2 |  |  |  |  |
|  |  | W | 2 | I | 2 |  |  |  |  |  |  |  |  |  |  |  |  | Y | 2 |  |  |  |  |
|  |  |  |  | M | 2 |  |  |  |  |  |  |  |  |  |  |  |  |  |  |  |  |  |  |
