# Supplementary material for: Proteolytic Characteristics of Cathepsin D Related to the Recognition and Cleavage of Its Target Proteins
Source: PLoS One. 2013 Jun 20;8(6):e65733. doi: 10.1371/journal.pone.0065733 (PMC3688724; doi:10.1371/journal.pone.0065733)
Supplement: Table S4 — The corresponding occurrence frequencies of the residues at each position in Figure 2A-left. (DOC) [file pone.0065733.s005.doc]

**Table S4. The corresponding occurrence frequencies of the residues at each position in Figure 2A-left.**

| **P6** | **%** | **P5** | **%** | **P4** | **%** | **P3** | **%** | **P2** | **%** | **P1** | **%** | **P1'** | **%** | **P2'** | **%** | **P3'** | **%** | **P4'** | **%** | **P5'** | **%** | **P6'** | **%** |
| --- | --- | --- | --- | --- | --- | --- | --- | --- | --- | --- | --- | --- | --- | --- | --- | --- | --- | --- | --- | --- | --- | --- | --- |
| E | 22 | L | 22 | I | 29 | I | 22 | E | 22 | L | 50 | V | 29 | L | 29 | A | 22 | L | 22 | D | 14 | E | 22 |
| A | 7 | E | 14 | A | 14 | K | 14 | R | 14 | F | 14 | P | 14 | E | 14 | E | 14 | S | 22 | Q | 14 | Q | 14 |
| R | 7 | Q | 14 | L | 14 | P | 14 | N | 14 | S | 14 | Y | 14 | V | 14 | G | 14 | P | 14 | L | 14 | S | 14 |
| N | 7 | K | 14 | M | 14 | S | 14 | I | 14 | E | 7 | R | 7 | A | 7 | S | 14 | A | 7 | A | 7 | T | 14 |
| D | 7 | R | 7 | G | 7 | R | 7 | M | 14 | N | 7 | E | 7 | R | 7 | Q | 7 | E | 7 | R | 7 | A | 7 |
| Q | 7 | C | 7 | P | 7 | N | 7 | D | 7 | W | 7 | Q | 7 | N | 7 | L | 7 | Q | 7 | E | 7 | N | 7 |
| G | 7 | G | 7 | Y | 7 | G | 7 | L | 7 |  |  | I | 7 | F | 7 | P | 7 | G | 7 | G | 7 | C | 7 |
| L | 7 | S | 7 | V | 7 | L | 7 | S | 7 |  |  | M | 7 | S | 7 | Y | 7 | F | 7 | M | 7 | P | 7 |
| M | 7 | T | 7 |  |  | T | 7 |  |  |  |  | W | 7 | T | 7 | V | 7 | W | 7 | S | 7 | Y | 7 |
| S | 7 |  |  |  |  |  |  |  |  |  |  |  |  |  |  |  |  |  |  | T | 7 |  |  |
| Y | 7 |  |  |  |  |  |  |  |  |  |  |  |  |  |  |  |  |  |  | V | 7 |  |  |
| V | 7 |  |  |  |  |  |  |  |  |  |  |  |  |  |  |  |  |  |  |  |  |  |  |
