# Supplementary material for: Proteolytic Characteristics of Cathepsin D Related to the Recognition and Cleavage of Its Target Proteins
Source: PLoS One. 2013 Jun 20;8(6):e65733. doi: 10.1371/journal.pone.0065733 (PMC3688724; doi:10.1371/journal.pone.0065733)
Supplement: Table S5 — The corresponding occurrence frequencies of the residues at each position in Figure 2A-right. (DOC) [file pone.0065733.s006.doc]

**Table S5. The corresponding occurrence frequencies of the residues at each position in Figure 2A-right.**

| **P6** | **%** | **P5** | **%** | **P4** | **%** | **P3** | **%** | **P2** | **%** | **P1** | **%** | **P1'** | **%** | **P2'** | **%** | **P3'** | **%** | **P4'** | **%** | **P5'** | **%** | **P6'** | **%** |
| --- | --- | --- | --- | --- | --- | --- | --- | --- | --- | --- | --- | --- | --- | --- | --- | --- | --- | --- | --- | --- | --- | --- | --- |
| S | 17 | S | 13 | V | 12 | I | 11 | S | 15 | L | 21 | I | 10 | V | 16 | L | 14 | A | 13 | A | 11 | S | 11 |
| L | 8 | N | 12 | A | 11 | L | 11 | E | 11 | F | 13 | A | 8 | E | 11 | S | 14 | E | 11 | V | 10 | E | 10 |
| T | 8 | A | 11 | I | 11 | V | 11 | L | 10 | Q | 8 | L | 8 | K | 10 | A | 11 | I | 10 | K | 8 | V | 8 |
| D | 7 | E | 8 | S | 8 | S | 10 | V | 10 | A | 7 | F | 8 | S | 10 | E | 10 | L | 9 | S | 8 | D | 7 |
| V | 7 | L | 8 | E | 7 | E | 8 | I | 8 | E | 7 | V | 8 | A | 8 | K | 8 | P | 9 | L | 7 | I | 7 |
| E | 6 | P | 6 | L | 7 | M | 8 | N | 8 | M | 6 | E | 7 | Q | 7 | Q | 6 | V | 9 | R | 6 | L | 7 |
| I | 6 | V | 6 | F | 6 | D | 5 | R | 6 | S | 6 | P | 7 | G | 7 | I | 6 | S | 6 | D | 6 | T | 7 |
| A | 5 | Q | 5 | N | 5 | Q | 5 | M | 6 | Y | 6 | S | 7 | L | 7 | M | 5 | R | 5 | E | 6 | Y | 7 |
| G | 5 | G | 5 | G | 5 | G | 5 | T | 5 | N | 5 | Y | 6 | I | 6 | R | 4 | Q | 5 | G | 6 | A | 5 |
| M | 5 | K | 5 | M | 5 | F | 5 | Y | 5 | V | 5 | K | 5 | R | 4 | G | 4 | K | 5 | N | 5 | R | 5 |
| P | 5 | R | 4 | T | 5 | T | 5 | Q | 4 | D | 4 | M | 5 | N | 2 | H | 4 | T | 5 | Q | 5 | N | 5 |
| N | 4 | D | 4 | P | 4 | K | 4 | G | 4 | C | 4 | R | 4 | D | 2 | T | 4 | N | 2 | Y | 5 | F | 5 |
| H | 4 | T | 4 | Y | 4 | P | 4 | D | 2 | K | 2 | N | 4 | M | 2 | D | 2 | D | 2 | I | 4 | K | 4 |
| F | 4 | I | 2 | R | 2 | A | 2 | K | 2 | W | 2 | Q | 4 | F | 2 | V | 2 | G | 2 | M | 4 | P | 4 |
| R | 2 | F | 2 | D | 2 | R | 2 | F | 2 | R | 1 | C | 2 | T | 2 | N | 1 | H | 2 | F | 4 | C | 2 |
| Q | 2 | C | 1 | Q | 2 | N | 2 | A | 1 | G | 1 | T | 2 | C | 1 | C | 1 | F | 2 | P | 4 | Q | 2 |
| K | 2 | H | 1 | K | 2 | Y | 2 | P | 1 | T | 1 | W | 2 | P | 1 | F | 1 | W | 2 | H | 1 | H | 2 |
| Y | 2 | M | 1 | W | 2 | W | 1 |  |  |  |  | D | 1 | Y | 1 | P | 1 | C | 1 | T | 1 | G | 1 |
| W | 1 | W | 1 |  |  |  |  |  |  |  |  | G | 1 |  |  | W | 1 | M | 1 | W | 1 | W | 1 |
|  |  | Y | 1 |  |  |  |  |  |  |  |  |  |  |  |  | Y | 1 |  |  |  |  |  |  |
