# Supplementary material for: Proteolytic Characteristics of Cathepsin D Related to the Recognition and Cleavage of Its Target Proteins
Source: PLoS One. 2013 Jun 20;8(6):e65733. doi: 10.1371/journal.pone.0065733 (PMC3688724; doi:10.1371/journal.pone.0065733)
Supplement: Table S6 — List of human CD cleavage sites in the substrate proteins used in this study. (DOC) [file pone.0065733.s007.doc]

**Table S6. List of human cathepsin D cleavage sites in substrate proteins used in this study.**

The residues surrounding the cleavage site from P6 to P6' are listed and the *arrow* indicates the cleavage site.

| **Protein** | **P6** | **P5** | **P4** | **P3** | **P2** | **P1** |  | **P1'** | **P2'** | **P3'** | **P4'** | **P5'** | **P6'** |
| --- | --- | --- | --- | --- | --- | --- | --- | --- | --- | --- | --- | --- | --- |
| BSA | A | H | R | F | K | D | ↓ | L | G | E | E | H | F |
|  | H | R | F | K | D | L | ↓ | G | E | E | H | F | K |
|  | E | E | H | F | K | G | ↓ | L | V | L | I | A | F |
|  | H | F | K | G | L | V | ↓ | L | I | A | F | S | Q |
|  | F | K | G | L | V | L | ↓ | I | A | F | S | Q | Y |
|  | L | I | A | F | S | Q | ↓ | Y | L | Q | Q | C | P |
|  | I | A | F | S | Q | Y | ↓ | L | Q | Q | C | P | F |
|  | A | F | S | Q | Y | L | ↓ | Q | Q | C | P | F | D |
|  | Q | Q | C | P | F | D | ↓ | E | H | V | K | L | V |
|  | Q | C | P | F | D | E | ↓ | H | V | K | L | V | N |
|  | C | P | F | D | E | H | ↓ | V | K | L | V | N | E |
|  | K | L | V | N | E | L | ↓ | T | E | F | A | K | T |
|  | V | N | E | L | T | E | ↓ | F | A | K | T | C | V |
|  | C | F | L | S | H | K | ↓ | D | D | S | P | D | L |
|  | D | D | S | P | D | L | ↓ | P | K | L | K | P | D |
|  | T | L | C | D | E | F | ↓ | K | A | D | E | K | K |
|  | K | F | W | G | K | Y | ↓ | L | Y | E | I | A | R |
|  | F | W | G | K | Y | L | ↓ | Y | E | I | A | R | R |
|  | G | K | Y | L | Y | E | ↓ | I | A | R | R | H | P |
|  | H | P | Y | F | Y | A | ↓ | P | E | L | L | Y | Y |
|  | F | Y | A | P | E | L | ↓ | L | Y | Y | A | N | K |
|  | Y | A | P | E | L | L | ↓ | Y | Y | A | N | K | Y |
|  | A | P | E | L | L | Y | ↓ | Y | A | N | K | Y | N |
|  | K | Y | N | G | V | F | ↓ | Q | E | C | C | Q | A |
|  | E | R | A | L | K | A | ↓ | W | S | V | A | R | L |
|  | K | A | W | S | V | A | ↓ | R | L | S | Q | K | F |
|  | W | S | V | A | R | L | ↓ | S | Q | K | F | P | K |
|  | R | L | S | Q | K | F | ↓ | P | K | A | E | F | V |
|  | K | F | P | K | A | E | ↓ | F | V | E | V | T | K |
|  | F | P | K | A | E | F | ↓ | V | E | V | T | K | L |
|  | K | A | E | F | V | E | ↓ | V | T | K | L | V | T |
|  | V | E | V | T | K | L | ↓ | V | T | D | L | T | K |
|  | T | K | L | V | T | D | ↓ | L | T | K | V | H | K |
|  | K | L | V | T | D | L | ↓ | T | K | V | H | K | E |
|  | A | E | V | E | K | D | ↓ | A | I | P | E | N | L |
|  | P | P | L | T | A | D | ↓ | F | A | E | D | K | D |
|  | Q | E | A | K | D | A | ↓ | F | L | G | S | F | L |
|  | F | L | G | S | F | L | ↓ | Y | E | Y | S | R | R |
|  | G | S | F | L | Y | E | ↓ | Y | S | R | R | H | P |
|  | Y | A | V | S | V | L | ↓ | L | R | L | A | K | E |
|  | E | Y | E | A | T | L | ↓ | E | E | C | C | A | K |
|  | C | Y | S | T | V | F | ↓ | D | K | L | K | H | L |
|  | D | E | P | Q | N | L | ↓ | I | K | Q | N | C | D |
|  | Q | N | C | D | Q | F | ↓ | E | K | L | G | E | Y |
|  | D | Q | F | E | K | L | ↓ | G | E | Y | G | F | Q |
|  | Y | G | F | Q | N | A | ↓ | L | I | V | R | Y | T |
|  | G | F | Q | N | A | L | ↓ | I | V | R | Y | T | R |
|  | N | A | L | I | V | R | ↓ | Y | T | R | K | V | P |
|  | A | L | I | V | R | Y | ↓ | T | R | K | V | P | Q |
|  | V | S | T | P | T | L | ↓ | V | E | V | S | R | S |
|  | A | L | T | P | D | E | ↓ | T | Y | V | P | K | A |
|  | L | T | P | D | E | T | ↓ | Y | V | P | K | A | F |
|  | Y | V | P | K | A | F | ↓ | D | E | K | L | F | T |
|  | A | F | D | E | K | L | ↓ | F | T | F | H | A | D |
|  | K | K | Q | T | A | L | ↓ | V | E | L | L | K | H |
|  | T | A | L | V | E | L | ↓ | L | K | H | K | P | K |
|  | A | L | V | E | L | L | ↓ | K | H | K | P | K | A |
|  | A | T | E | E | Q | L | ↓ | K | T | V | M | E | N |
|  | E | E | Q | L | K | T | ↓ | V | M | E | N | F | V |
|  | K | T | V | M | E | N | ↓ | F | V | A | F | V | D |
|  | T | V | M | E | N | F | ↓ | V | A | F | V | D | K |
|  | D | K | E | A | C | F | ↓ | A | V | E | G | P | K |
|  | C | F | A | V | E | G | ↓ | P | K | L | V | V | S |
|  | A | V | E | G | P | K | ↓ | L | V | V | S | T | Q |
|  | V | E | G | P | K | L | ↓ | V | V | S | T | Q | T |
|  | L | V | V | S | T | Q | ↓ | T | A | L | A |  |  |
|  | V | S | T | Q | T | A | ↓ | L | A |  |  |  |  |
| HSA |  |  |  |  |  | D | ↓ | A | H | K | S | E | V |
|  |  |  |  |  | D | A | ↓ | H | K | S | E | V | A |
|  | H | R | F | K | D | L | ↓ | G | E | E | N | F | K |
|  | D | L | G | E | E | N | ↓ | F | K | A | L | V | L |
|  | L | G | E | E | N | F | ↓ | K | A | L | V | L | I |
|  | E | E | N | F | K | A | ↓ | L | V | L | I | A | F |
|  | E | N | F | K | A | L | ↓ | V | L | I | A | F | A |
|  | Q | Y | L | Q | Q | C | ↓ | P | F | E | D | H | V |
|  | L | Q | Q | C | P | F | ↓ | E | D | H | V | K | L |
|  | C | P | F | E | D | H | ↓ | V | K | L | V | N | E |
|  | E | D | H | V | K | L | ↓ | V | N | E | V | T | E |
|  | V | N | E | V | T | E | ↓ | F | A | K | T | C | V |
|  | N | E | V | T | E | F | ↓ | A | K | T | C | V | A |
|  | V | M | C | T | A | F | ↓ | H | D | N | E | E | T |
|  | D | N | E | E | T | F | ↓ | L | K | K | Y | L | Y |
|  | K | F | G | E | R | A | ↓ | F | K | A | W | A | V |
|  | F | G | E | R | A | F | ↓ | K | A | W | A | V | A |
|  | R | A | F | K | A | W | ↓ | A | V | A | R | L | S |
|  | W | A | V | A | R | L | ↓ | S | Q | R | F | P | K |
|  | R | L | S | Q | R | F | ↓ | P | K | A | E | F | A |
|  | R | F | P | K | A | E | ↓ | F | A | E | V | S | K |
|  | F | P | K | A | E | F | ↓ | A | E | V | S | K | L |
|  | S | K | L | V | T | D | ↓ | L | T | K | V | H | T |
|  | K | L | V | T | D | L | ↓ | T | K | V | H | T | E |
|  | V | F | L | G | M | F | ↓ | L | Y | E | Y | A | R |
|  | G | M | F | L | Y | E | ↓ | Y | A | R | R | H | P |
|  | R | R | H | P | D | Y | ↓ | S | V | V | L | L | L |
|  | D | Y | S | V | V | L | ↓ | L | L | R | L | A | K |
|  | C | Y | A | K | V | F | ↓ | D | E | F | K | P | L |
|  | A | K | V | F | D | E | ↓ | F | K | P | L | V | E |
|  | K | V | F | D | E | F | ↓ | K | P | L | V | E | E |
|  | K | P | L | V | E | E | ↓ | P | Q | N | L | I | K |
|  | E | E | P | Q | N | L | ↓ | I | K | Q | N | C | E |
|  | K | Q | N | C | E | L | ↓ | F | E | Q | L | G | E |
|  | E | Q | L | G | E | Y | ↓ | K | F | Q | N | A | L |
|  | Y | K | F | Q | N | A | ↓ | L | L | V | R | Y | T |
|  | K | F | Q | N | A | L | ↓ | L | V | R | Y | T | K |
|  | F | Q | N | A | L | L | ↓ | V | R | Y | T | K | K |
|  | N | A | L | L | V | R | ↓ | Y | T | K | K | V | P |
|  | A | L | L | V | R | Y | ↓ | T | K | K | V | P | Q |
|  | V | S | T | P | T | L | ↓ | V | E | V | S | R | N |
|  | T | P | T | L | V | E | ↓ | V | S | R | N | L | G |
|  | P | C | F | S | A | L | ↓ | E | V | D | E | T | Y |
|  | F | S | A | L | E | V | ↓ | D | E | T | Y | V | P |
|  | S | A | L | E | V | D | ↓ | E | T | Y | V | P | K |
|  | A | L | E | V | D | E | ↓ | T | Y | V | P | K | E |
|  | Y | V | P | K | E | F | ↓ | N | A | E | T | F | T |
|  | E | F | N | A | E | T | ↓ | F | T | F | H | A | D |
|  | F | N | A | E | T | F | ↓ | T | F | H | A | D | I |
|  | N | A | E | T | F | T | ↓ | F | H | A | D | I | C |
|  | A | E | T | F | T | F | ↓ | H | A | D | I | C | T |
|  | T | A | L | V | E | L | ↓ | V | K | H | K | P | K |
|  | K | A | V | M | D | D | ↓ | F | A | A | F | V | E |
|  | A | V | M | D | D | F | ↓ | A | A | F | V | E | K |
|  | E | E | G | K | K | L | ↓ | V | A | A | S | Q | A |
| PSA |  |  | D | T | Y | K | ↓ | S | E | I | A | H | R |
|  | H | R | F | K | D | L | ↓ | G | E | Q | Y | F | K |
|  | K | D | L | G | E | Q | ↓ | Y | F | K | G | L | V |
|  | D | L | G | E | Q | Y | ↓ | F | K | G | L | V | L |
|  | E | Q | Y | F | K | G | ↓ | L | V | L | I | A | F |
|  | F | K | G | L | V | L | ↓ | I | A | F | S | Q | H |
|  | G | L | V | L | I | A | ↓ | F | S | Q | H | L | Q |
|  | C | P | Y | E | E | H | ↓ | V | K | L | V | R | E |
|  | Y | E | E | H | V | K | ↓ | L | V | R | E | V | T |
|  | E | E | H | V | K | L | ↓ | V | R | E | V | T | E |
|  | V | R | E | V | T | E | ↓ | F | A | K | T | C | V |
|  | R | E | V | T | E | F | ↓ | A | K | T | C | V | A |
|  | F | W | G | K | Y | L | ↓ | Y | E | I | A | R | R |
|  | H | P | Y | F | Y | A | ↓ | P | E | L | L | Y | Y |
|  | F | Y | A | P | E | L | ↓ | L | Y | Y | A | I | I |
|  | Y | A | P | E | L | L | ↓ | Y | Y | A | I | I | Y |
|  | A | P | E | L | L | Y | ↓ | Y | A | I | I | Y | K |
|  | P | E | L | L | Y | Y | ↓ | A | I | I | Y | K | D |
|  | I | I | Y | K | D | V | ↓ | F | S | E | C | C | Q |
|  | I | Y | K | D | V | F | ↓ | S | E | C | C | Q | A |
|  | F | K | A | W | S | L | ↓ | A | R | L | S | Q | R |
|  | W | S | L | A | R | L | ↓ | S | Q | R | F | P | K |
|  | S | L | A | R | L | S | ↓ | Q | R | F | P | K | A |
|  | A | R | L | S | Q | R | ↓ | F | P | K | A | D | F |
|  | S | Q | R | F | P | K | ↓ | A | D | F | T | E | I |
|  | Q | R | F | P | K | A | ↓ | D | F | T | E | I | S |
|  | R | F | P | K | A | D | ↓ | F | T | E | I | S | K |
|  | F | P | K | A | D | F | ↓ | T | E | I | S | K | I |
|  | K | A | D | F | T | E | ↓ | I | S | K | I | V | T |
|  | F | T | E | I | S | K | ↓ | I | V | T | D | L | A |
|  | K | I | V | T | D | L | ↓ | A | K | V | H | K | E |
|  | V | T | D | L | A | K | ↓ | V | H | K | E | C | C |
|  | A | K | R | D | E | L | ↓ | P | A | D | L | N | P |
|  | N | P | L | E | H | D | ↓ | F | V | E | D | K | E |
|  | Y | K | E | A | K | H | ↓ | V | F | L | G | T | F |
|  | V | F | L | G | T | F | ↓ | L | Y | E | Y | S | R |
|  | F | L | G | T | F | L | ↓ | Y | E | Y | S | R | R |
|  | D | Y | S | V | S | L | ↓ | L | L | R | I | A | K |
|  | Y | S | V | S | L | L | ↓ | L | R | I | A | K | I |
|  | I | A | K | I | Y | E | ↓ | A | T | L | E | D | C |
|  | K | I | Y | E | A | T | ↓ | L | E | D | C | C | A |
|  | C | Y | A | T | V | F | ↓ | D | K | F | Q | P | L |
|  | Y | A | T | V | F | D | ↓ | K | F | Q | P | L | V |
|  | L | V | D | E | P | K | ↓ | N | L | I | K | Q | N |
|  | V | D | E | P | K | N | ↓ | L | I | K | Q | N | C |
|  | D | E | P | K | N | L | ↓ | I | K | Q | N | C | E |
|  | E | L | F | E | K | L | ↓ | G | E | Y | G | F | Q |
|  | G | E | Y | G | F | Q | ↓ | N | A | L | I | V | R |
|  | E | Y | G | F | Q | N | ↓ | A | L | I | V | R | Y |
|  | Y | G | F | Q | N | A | ↓ | L | I | V | R | Y | T |
|  | G | F | Q | N | A | L | ↓ | I | V | R | Y | T | K |
|  | N | A | L | I | V | R | ↓ | Y | T | K | K | V | P |
|  | I | V | R | Y | T | K | ↓ | K | V | P | Q | V | S |
|  | V | S | T | P | T | L | ↓ | V | E | V | A | R | K |
|  | T | P | T | L | V | E | ↓ | V | A | R | K | L | G |
|  | R | P | C | F | S | A | ↓ | L | T | P | D | E | T |
|  | P | C | F | S | A | L | ↓ | T | P | D | E | T | Y |
|  | A | L | T | P | D | E | ↓ | T | Y | K | P | K | E |
|  | T | Y | K | P | K | E | ↓ | F | V | E | G | T | F |
|  | Y | K | P | K | E | F | ↓ | V | E | G | T | F | T |
|  | E | G | T | F | T | F | ↓ | H | A | D | L | C | T |
|  | K | K | Q | T | A | L | ↓ | V | E | L | L | K | H |
|  | Q | T | A | L | V | E | ↓ | L | L | K | H | K | P |
|  | T | A | L | V | E | L | ↓ | L | K | H | K | P | H |
|  | A | L | V | E | L | L | ↓ | K | H | K | P | H | A |
|  | A | T | E | E | Q | L | ↓ | R | T | V | L | G | N |
|  | T | E | E | Q | L | R | ↓ | T | V | L | G | N | F |
|  | Q | L | R | T | V | L | ↓ | G | N | F | A | A | F |
|  | T | V | L | G | N | F | ↓ | A | A | F | V | Q | K |
|  | L | G | N | F | A | A | ↓ | F | V | Q | K | C | C |
|  | D | H | E | A | C | F | ↓ | A | V | E | G | P | K |
|  | A | V | E | G | P | K | ↓ | F | V | I | E | I | R |
|  | V | E | G | P | K | F | ↓ | V | I | E | I | R | G |
|  | E | G | P | K | F | V | ↓ | I | E | I | R | G | I |
|  | E | I | R | G | I | L | ↓ | A |  |  |  |  |  |
| D-OVA | I | N | F | Q | T | A | ↓ | A | D | Q | A | R | E |
|  | G | L | W | E | K | A | ↓ | F | K | D | E | D | T |
|  | M | V | L | V | N | A | ↓ | I | V | F | K | G | L |
|  | I | A | I | M | S | A | ↓ | L | A | M | V | Y | L |
|  | V | D | S | Q | T | A | ↓ | M | V | L | V | N | A |
|  | T | S | V | L | M | A | ↓ | M | G | I | T | D | V |
|  | H | P | F | L | F | C | ↓ | I | K | H | I | A | T |
|  | E | N | I | F | Y | C | ↓ | P | I | A | I | M | S |
|  | P | E | Y | L | Q | C | ↓ | V | K | E | L | Y | R |
|  | A | E | A | G | V | D | ↓ | A | A | S | V | S | E |
|  | H | S | S | L | R | D | ↓ | I | L | N | Q | I | T |
|  | L | P | G | F | G | D | ↓ | S | I | E | A | Q | C |
|  | H | A | E | I | N | E | ↓ | A | G | R | E | V | V |
|  | S | S | N | V | M | E | ↓ | E | R | K | I | K | V |
|  | A | S | V | S | E | E | ↓ | F | R | A | D | H | P |
|  | P | F | R | V | T | E | ↓ | Q | E | S | K | P | V |
|  | S | N | V | M | E | E | ↓ | R | K | I | K | V | Y |
|  | I | N | S | W | V | E | ↓ | S | Q | T | N | G | I |
|  | D | H | P | F | L | F | ↓ | C | I | K | H | I | A |
|  | N | K | V | V | R | F | ↓ | D | K | L | P | G | F |
|  | E | S | I | I | N | F | ↓ | E | K | L | T | E | W |
|  | T | N | A | V | L | F | ↓ | F | G | R | C | V | S |
|  | V | N | A | I | V | F | ↓ | K | G | L | W | E | K |
|  | L | E | P | I | N | F | ↓ | Q | T | A | A | D | Q |
|  | T | Q | A | M | P | F | ↓ | R | V | T | E | Q | E |
|  | Y | Q | I | G | L | F | ↓ | R | V | A | S | M | A |
|  | N | D | V | Y | S | F | ↓ | S | L | A | S | R | L |
|  | G | I | T | D | V | F | ↓ | S | S | S | A | N | L |
|  | A | N | E | N | I | F | ↓ | Y | C | P | I | A | I |
|  | E | S | Q | T | N | G | ↓ | I | I | R | N | V | L |
|  | S | A | N | L | S | G | ↓ | I | S | S | A | E | S |
|  | S | A | E | S | L | K | ↓ | I | S | Q | A | V | H |
|  | T | E | Q | E | S | K | ↓ | P | V | Q | M | M | Y |
|  | V | Y | S | F | S | L | ↓ | A | S | R | L | Y | A |
|  | D | E | V | S | G | L | ↓ | E | Q | L | E | S | I |
|  | S | G | L | E | Q | L | ↓ | E | S | I | I | N | F |
|  | L | A | M | V | Y | L | ↓ | G | A | K | D | S | T |
|  | D | Q | A | R | E | L | ↓ | I | N | S | W | V | E |
|  | D | V | F | K | E | L | ↓ | K | V | H | H | A | N |
|  | S | S | A | E | S | L | ↓ | K | I | S | Q | A | V |
|  | M | S | M | L | V | L | ↓ | L | P | D | E | V | S |
|  | S | L | R | D | I | L | ↓ | N | Q | I | T | K | P |
|  | E | R | Y | P | I | L | ↓ | P | E | Y | L | Q | C |
|  | M | K | I | L | E | L | ↓ | P | F | A | S | G | T |
|  | S | M | L | V | L | L | ↓ | P | D | E | V | S | G |
|  | I | N | F | E | K | L | ↓ | T | E | W | T | S | S |
|  | Q | T | A | M | V | L | ↓ | V | N | A | I | V | F |
|  | G | T | M | S | M | L | ↓ | V | L | L | P | D | E |
|  | I | V | F | K | G | L | ↓ | W | E | K | A | F | K |
|  | S | L | A | S | R | L | ↓ | Y | A | E | E | R | Y |
|  | Q | C | V | K | E | L | ↓ | Y | R | G | G | L | E |
|  | S | G | T | M | S | M | ↓ | L | V | L | L | P | D |
|  | S | K | P | V | Q | M | ↓ | M | Y | Q | I | G | L |
|  | F | A | S | G | T | M | ↓ | S | M | L | V | L | L |
|  | D | S | Q | T | A | M | ↓ | V | L | V | N | A | I |
|  | K | P | V | Q | M | M | ↓ | Y | Q | I | G | L | F |
|  | G | L | E | P | I | N | ↓ | F | Q | T | A | A | D |
|  | T | R | T | Q | I | N | ↓ | K | V | V | R | F | D |
|  | N | G | I | I | R | N | ↓ | V | L | Q | P | S | S |
|  | E | W | T | S | S | N | ↓ | V | M | E | E | R | K |
|  | S | L | K | I | S | Q | ↓ | A | V | H | A | A | H |
|  | L | P | E | Y | L | Q | ↓ | C | V | K | E | L | Y |
|  | F | R | V | T | E | Q | ↓ | E | S | K | P | V | Q |
|  | R | D | I | L | N | Q | ↓ | I | T | K | P | N | D |
|  | V | Q | M | M | Y | Q | ↓ | I | G | L | F | R | V |
|  | V | S | G | L | E | Q | ↓ | L | E | S | I | I | N |
|  | S | S | V | D | S | Q | ↓ | T | A | M | V | L | V |
|  | T | N | G | I | I | R | ↓ | N | V | L | Q | P | S |
|  | L | S | G | I | S | S | ↓ | A | E | S | L | K | I |
|  | P | N | D | V | Y | S | ↓ | F | S | L | A | S | R |
|  | D | V | Y | S | F | S | ↓ | L | A | S | R | L | Y |
|  | T | E | W | T | S | S | ↓ | N | V | M | E | E | R |
|  | R | E | L | I | N | S | ↓ | W | V | E | S | Q | T |
|  | F | K | D | E | D | T | ↓ | Q | A | M | P | F | R |
|  | L | V | N | A | I | V | ↓ | F | K | G | L | W | E |
|  | M | G | I | T | D | V | ↓ | F | S | S | S | A | N |
|  | W | T | S | S | N | V | ↓ | M | E | E | R | K | I |
|  | S | A | L | A | M | V | ↓ | Y | L | G | A | K | D |
|  | V | F | K | G | L | W | ↓ | E | K | A | F | K | D |
|  | E | L | I | N | S | W | ↓ | V | E | S | Q | T | N |
|  | L | A | S | R | L | Y | ↓ | A | E | E | R | Y | P |
|  | A | L | A | M | V | Y | ↓ | L | G | A | K | D | S |
|  | P | I | L | P | E | Y | ↓ | L | Q | C | V | K | E |
|  | Y | A | E | E | R | Y | ↓ | P | I | L | P | E | Y |
|  | K | P | N | D | V | Y | ↓ | S | F | S | L | A | S |
| TF | I | R | A | I | A | A | ↓ | N | E | A | D | A | V |
|  | I | A | A | N | E | A | ↓ | D | A | V | T | L | D |
|  | A | A | N | E | A | D | ↓ | A | V | T | L | D | A |
|  | A | D | A | V | T | L | ↓ | D | A | G | L | V | Y |
|  | D | A | V | T | L | D | ↓ | A | G | L | V | Y | D |
|  | V | T | L | D | A | G | ↓ | L | V | Y | D | A | Y |
|  | D | A | G | L | V | Y | ↓ | D | A | Y | L | A | P |
|  | A | G | L | V | Y | D | ↓ | A | Y | L | A | P | N |
|  | G | L | V | Y | D | A | ↓ | Y | L | A | P | N | N |
|  | L | V | Y | D | A | Y | ↓ | L | A | P | N | N | L |
|  | L | K | P | V | V | A | ↓ | E | F | Y | G | S | K |
|  | K | P | V | V | A | E | ↓ | F | Y | G | S | K | E |
|  | P | V | V | A | E | F | ↓ | Y | G | S | K | E | D |
|  | V | V | A | E | F | Y | ↓ | G | S | K | E | D | P |
|  | Y | G | S | K | E | D | ↓ | P | Q | T | F | Y | Y |
|  | E | D | P | Q | T | F | ↓ | Y | Y | A | V | A | V |
|  | D | P | Q | T | F | Y | ↓ | Y | A | V | A | V | V |
|  | P | Q | T | F | Y | Y | ↓ | A | V | A | V | V | K |
|  | Q | T | F | Y | Y | A | ↓ | V | A | V | V | K | K |
|  | D | S | G | F | Q | M | ↓ | N | Q | L | R | G | K |
|  | K | D | G | A | G | D | ↓ | V | A | F | V | K | H |
|  | G | A | G | D | V | A | ↓ | F | V | K | H | S | T |
|  | A | G | D | V | A | F | ↓ | V | K | H | S | T | I |
|  | K | H | S | T | I | F | ↓ | E | N | L | A | N | K |
|  | S | T | I | F | E | N | ↓ | L | A | N | K | A | D |
|  | T | I | F | E | N | L | ↓ | A | N | K | A | D | R |
|  | R | D | Q | Y | E | L | ↓ | L | C | L | D | N | T |
|  | D | Q | Y | E | L | L | ↓ | C | L | D | N | T | R |
|  | K | E | D | L | I | W | ↓ | E | L | L | N | Q | A |
|  | E | D | L | I | W | E | ↓ | L | L | N | Q | A | Q |
|  | D | L | I | W | E | L | ↓ | L | N | Q | A | Q | E |
|  | L | I | W | E | L | L | ↓ | N | Q | A | Q | E | H |
|  | W | E | L | L | N | Q | ↓ | A | Q | E | H | F | G |
|  | S | K | E | F | Q | L | ↓ | F | S | S | P | H | G |
|  | H | G | K | D | L | L | ↓ | F | K | D | S | A | H |
|  | D | S | A | H | G | F | ↓ | L | K | V | P | P | R |
|  | A | K | M | Y | L | G | ↓ | Y | E | Y | V | T | A |
|  | M | Y | L | G | Y | E | ↓ | Y | V | T | A | I | R |
|  | Y | E | Y | V | T | A | ↓ | I | R | N | L | R | E |
|  | T | A | I | R | N | L | ↓ | R | E | G | T | C | P |
|  | I | M | N | G | E | A | ↓ | D | A | M | S | L | D |
|  | G | E | A | D | A | M | ↓ | S | L | D | G | G | F |
|  | S | L | D | G | G | F | ↓ | V | Y | I | A | G | K |
|  | E | K | G | D | V | A | ↓ | F | V | K | H | Q | T |
|  | D | V | A | F | V | K | ↓ | H | Q | T | V | P | Q |
|  | P | D | P | W | A | K | ↓ | N | L | N | E | K | D |
|  | P | W | A | K | N | L | ↓ | N | E | K | D | Y | E |
|  | S | G | N | F | C | L | ↓ | F | R | S | E | T | K |
|  | S | E | T | K | D | L | ↓ | L | F | R | D | D | T |
|  | E | T | K | D | L | L | ↓ | F | R | D | D | T | V |
|  | D | D | T | V | C | L | ↓ | A | K | L | H | D | R |
|  | K | Y | L | G | E | E | ↓ | Y | V | K | A | V | G |
|  | K | A | V | G | N | L | ↓ | R | K | S | T | S |  |
| HB | A | N | V | K | A | A | ↓ | W | G | K | V | G | G |
|  | N | V | K | A | A | W | ↓ | G | K | V | G | G | Q |
|  | V | K | A | A | W | G | ↓ | K | V | G | G | Q | A |
|  | K | V | G | G | Q | A | ↓ | G | A | H | G | A | E |
|  | V | G | G | Q | A | G | ↓ | A | H | G | A | E | A |
|  | A | H | G | A | E | A | ↓ | L | E | R | M | F | L |
|  | E | A | L | E | R | M | ↓ | F | L | G | F | P | T |
|  | A | L | E | R | M | F | ↓ | L | G | F | P | T | T |
|  | P | T | T | K | T | Y | ↓ | F | P | H | F | N | L |
|  | T | Y | F | P | H | F | ↓ | N | L | S | H | G | S |
|  | Y | F | P | H | F | N | ↓ | L | S | H | G | S | D |
|  | F | N | L | S | H | G | ↓ | S | D | Q | V | K | A |
|  | L | S | H | G | S | D | ↓ | Q | V | K | A | H | G |
|  | S | A | L | S | D | L | ↓ | H | A | H | K | L | R |
|  | H | K | L | R | V | D | ↓ | P | V | N | F | K | L |
|  | V | D | P | V | N | F | ↓ | K | L | L | S | H | C |
|  | L | L | S | H | C | L | ↓ | L | V | T | L | A | A |
|  | L | S | H | C | L | L | ↓ | V | T | L | A | A | H |
|  | H | C | L | L | V | T | ↓ | L | A | A | H | H | P |
|  | C | L | L | V | T | L | ↓ | A | A | H | H | P | D |
|  | T | L | A | A | H | H | ↓ | P | D | D | F | N | P |
|  | A | A | H | H | P | D | ↓ | D | F | N | P | S | V |
|  | A | H | H | P | D | D | ↓ | F | N | P | S | V | H |
|  | F | N | P | S | V | H | ↓ | A | S | L | D | K | F |
|  | N | P | S | V | H | A | ↓ | S | L | D | K | F | L |
|  | S | V | H | A | S | L | ↓ | D | K | F | L | A | N |
|  | V | H | A | S | L | D | ↓ | K | F | L | A | N | V |
|  | A | S | L | D | K | F | ↓ | L | A | N | V | S | T |
|  | S | L | D | K | F | L | ↓ | A | N | V | S | T | V |
|  | L | D | K | F | L | A | ↓ | N | V | S | T | V | L |
|  | D | K | F | L | A | N | ↓ | V | S | T | V | L | T |
|  | N | V | S | T | V | L | ↓ | T | S | K | Y | R |  |
|  | K | E | A | V | L | G | ↓ | L | W | G | K | V | N |
|  | E | A | V | L | G | L | ↓ | W | G | K | V | N | V |
|  | A | V | L | G | L | W | ↓ | G | K | V | N | V | D |
|  | L | W | G | K | V | N | ↓ | V | D | E | V | G | G |
|  | W | G | K | V | N | V | ↓ | D | E | V | G | G | E |
|  | G | K | V | N | V | D | ↓ | E | V | G | G | E | A |
|  | K | V | N | V | D | E | ↓ | V | G | G | E | A | L |
|  | G | E | A | L | G | R | ↓ | L | L | V | V | Y | P |
|  | E | A | L | G | R | L | ↓ | L | V | V | Y | P | W |
|  | A | L | G | R | L | L | ↓ | V | V | Y | P | W | T |
|  | P | W | T | Q | R | F | ↓ | F | E | S | F | G | D |
|  | V | D | P | E | N | F | ↓ | R | L | L | G | N | V |
|  | N | F | R | L | L | G | ↓ | N | V | I | V | V | V |
|  | L | G | N | V | I | V | ↓ | V | V | L | A | R | R |
|  | V | I | V | V | V | L | ↓ | A | R | R | L | G | H |
|  | P | N | V | Q | A | A | ↓ | F | Q | K | V | V | A |
|  | K | V | V | A | G | V | ↓ | A | N | A | L | A | H |
| AKR1A1 | A | A | S | C | V | L | ↓ | L | H | T | G | Q | K |
|  | M | P | L | I | G | L | ↓ | G | T | W | K | S | E |
|  | L | I | G | L | G | T | ↓ | W | K | S | E | P | G |
|  | I | G | L | G | T | W | ↓ | K | S | E | P | G | Q |
|  | Y | A | L | S | V | G | ↓ | Y | R | H | I | D | C |
|  | Y | R | H | I | D | C | ↓ | A | A | I | Y | G | N |
|  | P | E | I | G | E | A | ↓ | L | K | E | D | V | G |
|  | E | I | G | E | A | L | ↓ | K | E | D | V | G | P |
|  | V | P | R | E | E | L | ↓ | F | V | T | S | K | L |
|  | P | R | E | E | L | F | ↓ | V | T | S | K | L | W |
|  | F | V | T | S | K | L | ↓ | W | N | T | K | H | H |
|  | A | L | R | K | T | L | ↓ | A | D | L | Q | L | E |
|  | Q | I | D | D | I | L | ↓ | S | V | A | S | V | R |
|  | V | R | P | A | V | L | ↓ | Q | V | E | C | H | P |
|  | L | A | Q | N | E | L | ↓ | I | A | H | C | Q | A |
|  | R | G | L | E | V | T | ↓ | A | Y | S | P | L | G |
|  | P | D | E | P | V | L | ↓ | L | E | E | P | V | V |
|  | E | E | P | V | V | L | ↓ | A | L | A | E | K | Y |
|  | E | P | V | V | L | A | ↓ | L | A | E | K | Y | G |
|  | P | V | V | L | A | L | ↓ | A | E | K | Y | G | R |
|  | S | P | A | Q | I | L | ↓ | L | R | W | Q | V | Q |
|  | Q | N | I | K | V | F | ↓ | D | F | T | F | S | P |
|  | N | I | K | V | F | D | ↓ | F | T | F | S | P | E |
|  | E | E | M | K | Q | L | ↓ | N | A | L | N | K | N |
|  | A | L | N | K | N | W | ↓ | R | Y | I | V | P | M |
|  | N | K | N | W | R | Y | ↓ | I | V | P | M | L | T |
| AKR1B10 |  |  |  | M | A | T | ↓ | F | V | E | L | S | T |
|  |  |  | M | A | T | F | ↓ | V | E | L | S | T | K |
|  | M | A | T | F | V | E | ↓ | L | S | T | K | A | K |
|  | A | T | F | V | E | L | ↓ | S | T | K | A | K | M |
|  | M | P | I | V | G | L | ↓ | G | T | W | K | S | P |
|  | I | V | G | L | G | T | ↓ | W | K | S | P | L | G |
|  | E | A | V | K | V | A | ↓ | I | D | A | G | Y | R |
|  | V | A | I | D | A | G | ↓ | Y | R | H | I | D | C |
|  | G | Y | R | H | I | D | ↓ | C | A | Y | V | Y | Q |
|  | R | H | I | D | C | A | ↓ | Y | V | Y | Q | N | E |
|  | H | I | D | C | A | Y | ↓ | V | Y | Q | N | E | H |
|  | Y | V | Y | Q | N | E | ↓ | H | E | V | G | E | A |
|  | H | E | V | G | E | A | ↓ | I | Q | E | K | I | Q |
|  | G | E | A | I | Q | E | ↓ | K | I | Q | E | K | A |
|  | E | A | I | Q | E | K | ↓ | I | Q | E | K | A | V |
|  | E | K | I | Q | E | K | ↓ | A | V | K | R | E | D |
|  | V | K | R | E | D | L | ↓ | F | I | V | S | K | L |
|  | K | R | E | D | L | F | ↓ | I | V | S | K | L | W |
|  | D | L | F | I | V | S | ↓ | K | L | W | P | T | F |
|  | K | L | W | P | T | F | ↓ | F | E | R | P | L | V |
|  | E | K | T | L | K | D | ↓ | L | K | L | S | Y | L |
|  | L | K | D | L | K | L | ↓ | S | Y | L | D | V | Y |
|  | Y | L | D | V | Y | L | ↓ | I | H | W | P | Q | G |
|  | S | G | D | D | L | F | ↓ | P | K | D | D | K | G |
|  | K | D | D | K | G | N | ↓ | A | I | G | G | K | A |
|  | N | A | I | G | G | K | ↓ | A | T | F | L | D | A |
|  | G | K | A | T | F | L | ↓ | D | A | W | E | A | M |
|  | A | T | F | L | D | A | ↓ | W | E | A | M | E | E |
|  | D | A | W | E | A | M | ↓ | E | E | L | V | D | E |
|  | W | E | A | M | E | E | ↓ | L | V | D | E | G | L |
|  | E | A | M | E | E | L | ↓ | V | D | E | G | L | V |
|  | E | G | L | V | K | A | ↓ | L | G | V | S | N | F |
|  | G | L | V | K | A | L | ↓ | G | V | S | N | F | S |
|  | A | L | G | V | S | N | ↓ | F | S | H | F | Q | I |
|  | L | G | V | S | N | F | ↓ | S | H | F | Q | I | E |
|  | F | Q | I | E | K | L | ↓ | L | N | K | P | G | L |
|  | Y | K | P | V | T | N | ↓ | Q | V | E | C | H | P |
|  | K | P | V | T | N | Q | ↓ | V | E | C | H | P | Y |
|  | L | T | Q | E | K | L | ↓ | I | Q | Y | C | H | S |
|  | K | G | I | T | V | T | ↓ | A | Y | S | P | L | G |
|  | G | I | T | V | T | A | ↓ | Y | S | P | L | G | S |
|  | P | E | D | P | S | L | ↓ | L | E | D | P | K | I |
|  | P | S | L | L | E | D | ↓ | P | K | I | K | E | I |
|  | D | P | K | I | K | E | ↓ | I | A | A | K | H | K |
|  | T | A | A | Q | V | L | ↓ | I | R | F | H | I | Q |
|  | Q | V | L | I | R | F | ↓ | H | I | Q | R | N | V |
|  | F | H | I | Q | R | N | ↓ | V | I | V | I | P | K |
|  | P | A | R | I | V | E | ↓ | N | I | Q | V | F | D |
|  | A | R | I | V | E | N | ↓ | I | Q | V | F | D | F |
|  | R | I | V | E | N | I | ↓ | Q | V | F | D | F | K |
|  | E | N | I | Q | V | F | ↓ | D | F | K | L | S | D |
|  | N | I | Q | V | F | D | ↓ | F | K | L | S | D | E |
|  | I | Q | V | F | D | F | ↓ | K | L | S | D | E | E |
|  | L | S | D | E | E | M | ↓ | A | T | I | L | S | F |
|  | S | D | E | E | M | A | ↓ | T | I | L | S | F | N |
|  | D | E | E | M | A | T | ↓ | I | L | S | F | N | R |
|  | E | M | A | T | I | L | ↓ | S | F | N | R | N | W |
|  | A | T | I | L | S | F | ↓ | N | R | N | W | R | A |
|  | L | S | F | N | R | N | ↓ | W | R | A | C | N | V |
|  | S | F | N | R | N | W | ↓ | R | A | C | N | V | L |
| AKR1C1 | F | M | P | V | L | G | ↓ | F | G | T | Y | A | P |
|  | M | P | V | L | G | F | ↓ | G | T | Y | A | P | A |
|  | L | G | F | G | T | Y | ↓ | A | P | A | E | V | P |
|  | G | F | G | T | Y | A | ↓ | P | A | E | V | P | K |
|  | L | E | A | T | K | L | ↓ | A | I | E | A | G | F |
|  | L | A | I | E | A | G | ↓ | F | R | H | I | D | S |
|  | H | L | Y | N | N | E | ↓ | E | Q | V | G | L | A |
|  | N | E | E | Q | V | G | ↓ | L | A | I | R | S | K |
|  | K | R | E | D | I | F | ↓ | Y | T | S | K | L | W |
|  | R | G | V | V | V | L | ↓ | A | K | S | Y | N | E |
|  | N | V | Q | V | F | E | ↓ | F | Q | L | T | S | E |
|  | V | Q | V | F | E | F | ↓ | Q | L | T | S | E | E |
|  | V | F | E | F | Q | L | ↓ | T | S | E | E | M | K |
|  | D | G | L | N | R | N | ↓ | V | R | Y | L | T | L |
|  | N | R | N | V | R | Y | ↓ | L | T | L | D | I | F |
| AKR1C3 | F | M | P | V | L | G | ↓ | F | G | T | Y | A | P |
|  | L | G | F | G | T | Y | ↓ | A | P | P | E | V | P |
|  | P | R | S | K | A | L | ↓ | E | V | T | K | L | A |
|  | L | E | V | T | K | L | ↓ | A | I | E | A | G | F |
|  | L | A | I | E | A | G | ↓ | F | R | H | I | D | S |
|  | N | E | E | Q | V | G | ↓ | L | A | I | R | S | K |
|  | K | R | E | D | I | F | ↓ | Y | T | S | K | L | W |
|  | Y | V | D | L | Y | L | ↓ | I | H | S | P | M | S |
|  | G | K | V | I | F | D | ↓ | I | V | D | L | C | T |
|  | R | R | Q | L | E | M | ↓ | I | L | N | K | P | G |
|  | Y | K | P | V | C | N | ↓ | Q | V | E | C | H | P |
|  | R | S | K | L | L | D | ↓ | F | C | K | S | K | D |
|  | S | K | D | I | V | L | ↓ | V | A | Y | S | A | L |
|  | P | N | S | P | V | L | ↓ | L | E | D | P | V | L |
|  | P | A | L | I | A | L | ↓ | R | Y | Q | L | Q | R |
|  | R | G | V | V | V | L | ↓ | A | K | S | Y | N | E |
|  | N | V | Q | V | F | E | ↓ | F | Q | L | T | A | E |
|  | V | Q | V | F | E | F | ↓ | Q | L | T | A | E | D |
|  | K | A | I | D | G | L | ↓ | D | R | N | L | H | Y |
|  | R | N | L | H | Y | F | ↓ | N | S | D | S | F | A |
|  | F | N | S | D | S | F | ↓ | A | S | H | P | N | Y |
|  | Y | P | Y | S | D | E | ↓ | Y |  |  |  |  |  |
| GST-M1 | L | D | F | P | N | L | ↓ | P | Y | L | I | D | G |
|  | K | I | T | Q | S | N | ↓ | A | I | L | R | Y | L |
|  | E | R | I | R | A | D | ↓ | I | V | E | N | Q | V |
|  | D | T | R | M | Q | L | ↓ | I | M | L | C | Y | N |
|  | P | E | K | M | K | L | ↓ | Y | S | E | F | L | G |
|  | R | P | W | F | A | G | ↓ | D | K | V | T | Y | V |
| GST-P1 | C | E | A | M | R | M | ↓ | L | L | A | D | Q | G |
|  | E | A | M | R | M | L | ↓ | L | A | D | Q | G | Q |
|  | Q | S | W | K | E | E | ↓ | V | V | T | I | D | T |
|  | E | A | A | Q | M | D | ↓ | M | V | N | D | G | V |
|  | D | G | V | E | D | L | ↓ | R | G | K | Y | V | T |
|  | D | L | R | G | K | Y | ↓ | V | T | L | I | Y | T |
|  | G | K | Y | V | T | L | ↓ | I | Y | T | N | Y | E |
|  | K | P | F | E | T | L | ↓ | L | S | Q | N | Q | G |
|  | Q | G | G | K | A | F | ↓ | I | V | G | D | Q | I |
|  | F | I | V | G | D | Q | ↓ | I | S | F | A | D | Y |
|  | G | D | Q | I | S | F | ↓ | A | D | Y | N | L | L |
|  | G | C | L | D | N | F | ↓ | P | L | L | S | A | Y |
|  | P | L | L | S | A | Y | ↓ | V | A | R | L | S | A |
| GST-A3 | P | I | R | W | L | L | ↓ | A | A | A | G | V | E |
|  | V | E | F | E | E | K | ↓ | F | L | K | T | R | D |
|  | E | F | E | E | K | F | ↓ | L | K | T | R | D | D |
| CD | P | E | V | L | K | N | ↓ | Y | M | D | A | Q | Y |
|  | E | V | L | K | N | Y | ↓ | M | D | A | Q | Y | Y |
|  | Y | M | D | A | Q | Y | ↓ | Y | G | E | I | G | I |
|  | K | N | G | T | S | F | ↓ | D | I | H | Y | G | S |
|  | S | G | S | L | S | G | ↓ | Y | L | S | Q | D | T |
|  | G | S | L | S | G | Y | ↓ | L | S | Q | D | T | V |
|  | G | V | K | V | E | R | ↓ | Q | V | F | G | E | A |
|  | V | E | R | Q | V | F | ↓ | G | E | A | T | K | Q |
|  | Q | P | G | I | T | F | ↓ | I | A | A | K | F | D |
|  | F | D | G | I | L | G | ↓ | M | A | Y | P | R | I |
|  | I | L | G | M | A | Y | ↓ | P | R | I | S | V | N |
|  | P | R | I | S | V | N | ↓ | N | V | L | P | V | F |
|  | S | V | N | N | V | L | ↓ | P | V | F | D | N | L |
|  | P | V | F | D | N | L | ↓ | M | Q | Q | K | L | V |
|  | K | L | V | D | Q | N | ↓ | I | F | S | F | Y | L |
|  | V | D | Q | N | I | F | ↓ | S | F | Y | L | S | R |
|  | D | Q | N | I | F | S | ↓ | F | Y | L | S | R | D |
|  | Q | N | I | F | S | F | ↓ | Y | L | S | R | D | P |
|  | N | I | F | S | F | Y | ↓ | L | S | R | D | P | D |
|  | I | F | S | F | Y | L | ↓ | S | R | D | P | D | A |
|  | Q | P | G | G | E | L | ↓ | M | L | G | G | T | D |
|  | P | G | G | E | L | M | ↓ | L | G | G | T | D | S |
|  | G | G | E | L | M | L | ↓ | G | G | T | D | S | K |
|  | G | G | T | D | S | K | ↓ | Y | Y | K | G | S | L |
|  | G | T | D | S | K | Y | ↓ | Y | K | G | S | L | S |
|  | D | T | G | T | S | L | ↓ | M | V | G | P | V | D |
|  | V | G | P | V | D | E | ↓ | V | R | E | L | Q | K |
|  | V | D | E | V | R | E | ↓ | L | Q | K | A | I | G |
|  | D | E | V | R | E | L | ↓ | Q | K | A | I | G | A |
|  | V | R | E | L | Q | K | ↓ | A | I | G | A | V | P |
|  | R | E | L | Q | K | A | ↓ | I | G | A | V | P | L |
|  | P | L | I | Q | G | E | ↓ | Y | M | I | P | C | E |
|  | L | P | A | I | T | L | ↓ | K | L | G | G | K | G |
|  | K | L | S | P | E | D | ↓ | Y | T | L | K | V | S |
|  | P | E | D | Y | T | L | ↓ | K | V | S | Q | A | G |
|  | V | F | I | G | R | Y | ↓ | Y | T | V | F | D | R |
|  | R | Y | Y | T | V | F | ↓ | D | R | D | N | N | R |
|  | N | N | R | V | G | F | ↓ | A | E | A | A | R | L |
|  | G | F | A | E | A | A | ↓ | R | L |  |  |  |  |
|  | F | A | E | A | A | R | ↓ | L |  |  |  |  |  |
