# Supplementary material for: Proteolytic Characteristics of Cathepsin D Related to the Recognition and Cleavage of Its Target Proteins
Source: PLoS One. 2013 Jun 20;8(6):e65733. doi: 10.1371/journal.pone.0065733 (PMC3688724; doi:10.1371/journal.pone.0065733)
Supplement: Table S7 — Comparison of the T-HSNs among the substrates used in this study (upper panel) and resistant proteins verified in this study (lower panel). (DOC) [file pone.0065733.s008.doc]

**Table S7. Comparison of T-HSNs among substrates used in this study (*upper panel*) and resistant proteins verified in this study (*lower panel*).**

The average, median and STDEVP of T-HSN in each protein are calculated and listed below.

| **T-HSN** | **BSA** | **AKR1A1** | **AKR1B10** | **AKR1C1** | **AKR1C3** | **GSTs** | **HB** | **TF** |
| --- | --- | --- | --- | --- | --- | --- | --- | --- |
| Average | 0.6 | 0.95 | 0.75 | 0.89 | 0.79 | 0.88 | 0.73 | 0.72 |
| Median | 0.47 | 0.93 | 0.89 | 0.95 | 0.93 | 1.16 | 0.97 | 0.51 |
| STDEVP | 1.67 | 1.35 | 1.63 | 1.39 | 1.43 | 1.56 | 1.55 | 1.53 |
| **T-HSN** | **OVA** | **Chymotrypsin** | **Trypsin** | **Trx** | **NDKA** | **FABP5** | **COTL1** | **CD** |
| Average | 0.67 | 0.76 | 0.69 | 0.71 | 0.69 | 0.43 | 0.64 | 0.88 |
| Median | 0.84 | 0.84 | 0.84 | 0.83 | 0.25 | 0.16 | 0.42 | 1.16 |
| STDEVP | 1.52 | 1.42 | 1.5 | 1.46 | 1.68 | 1.39 | 1.62 | 1.42 |
